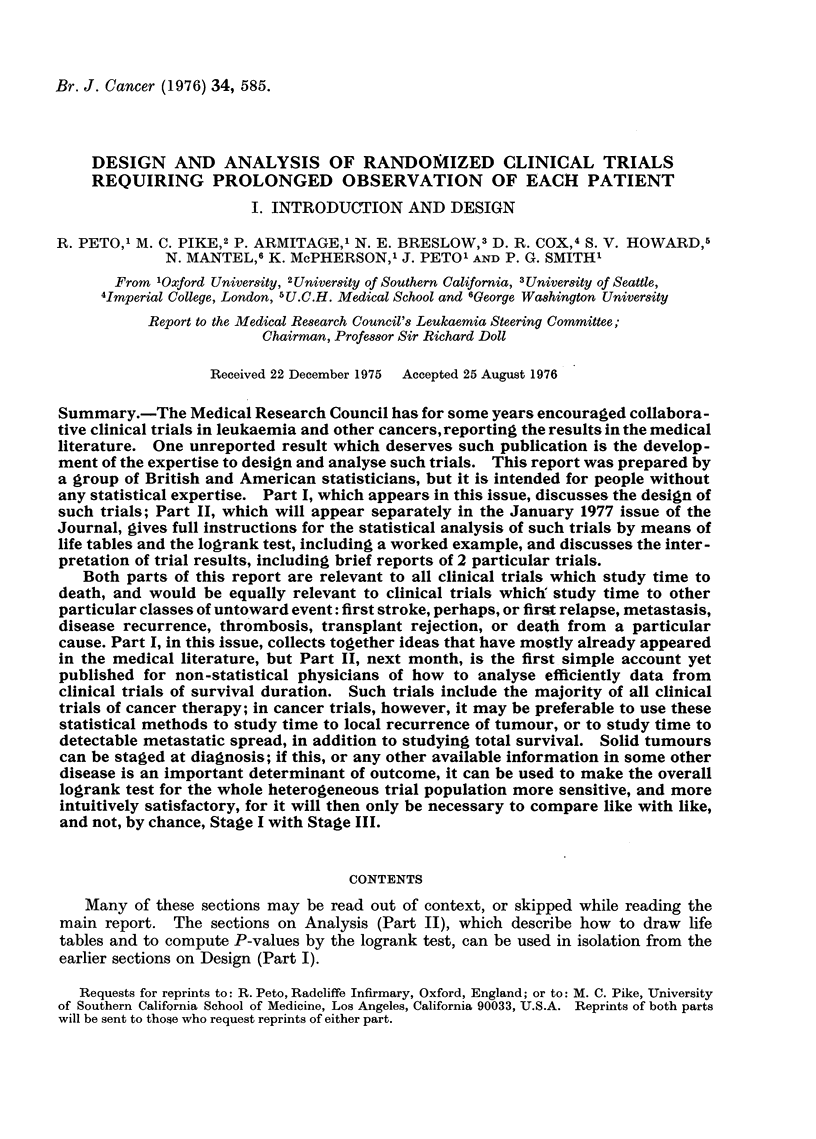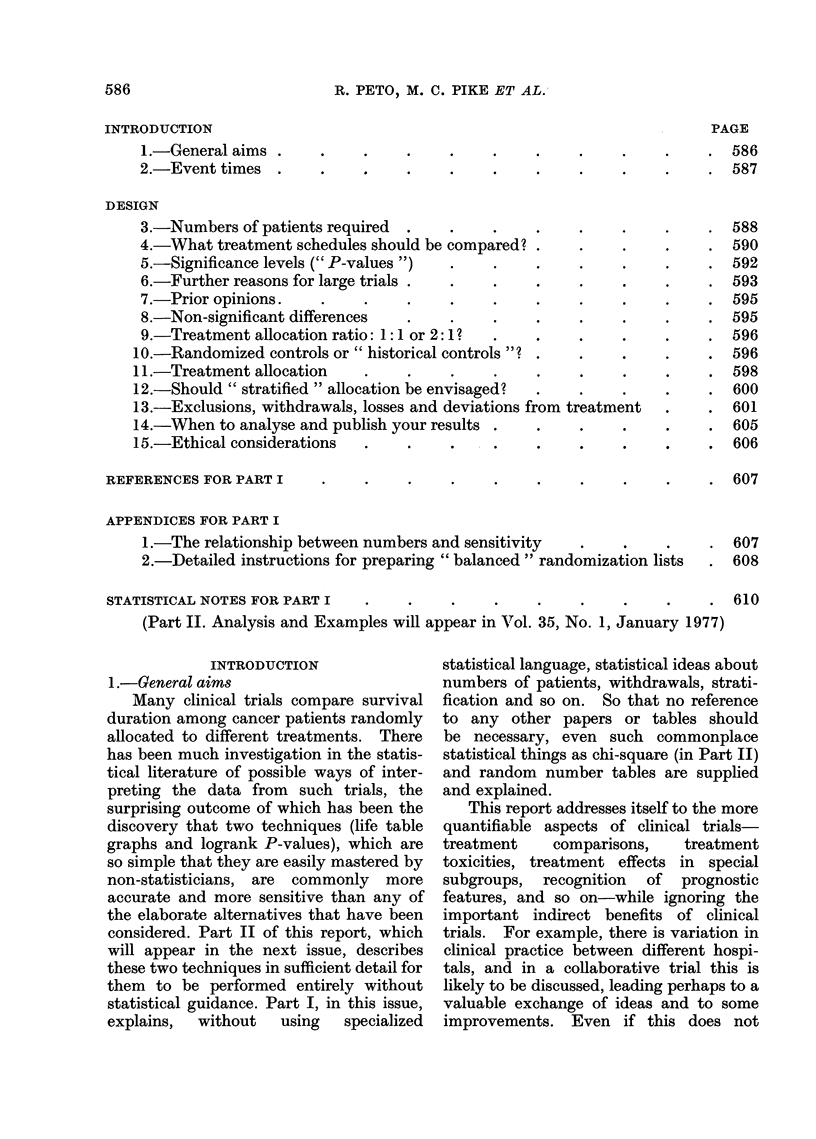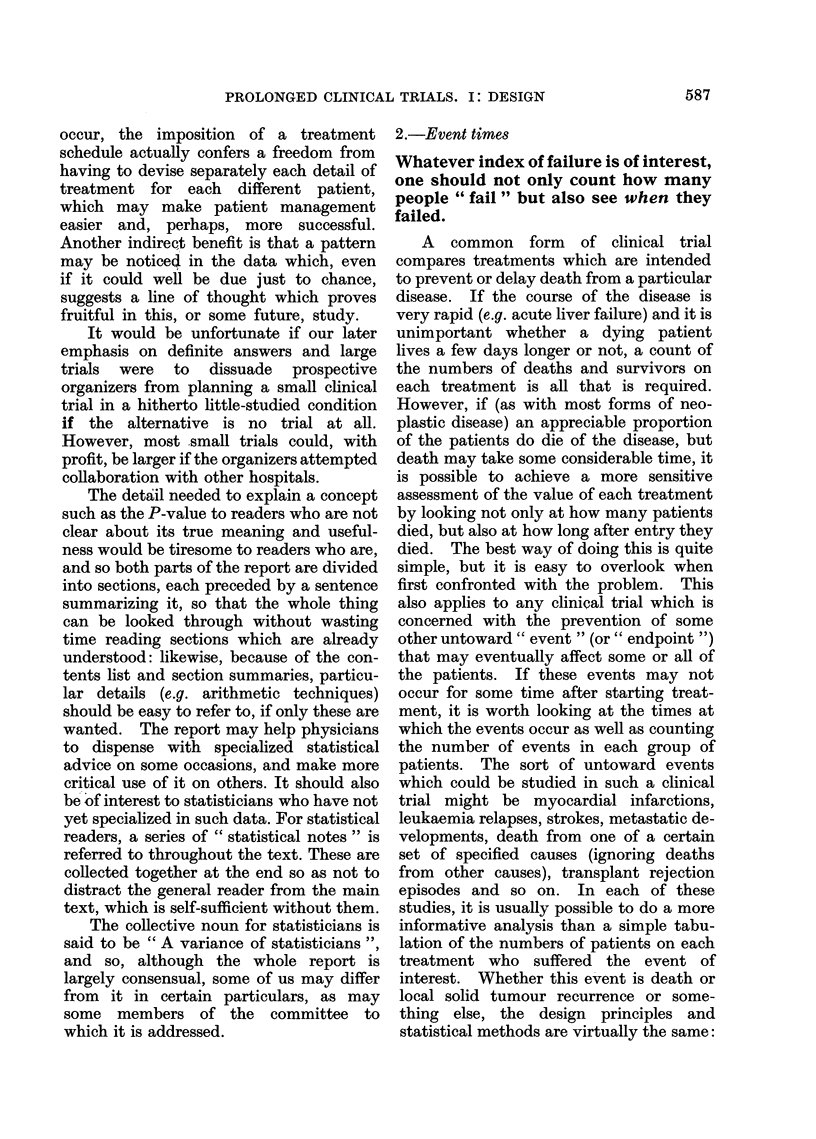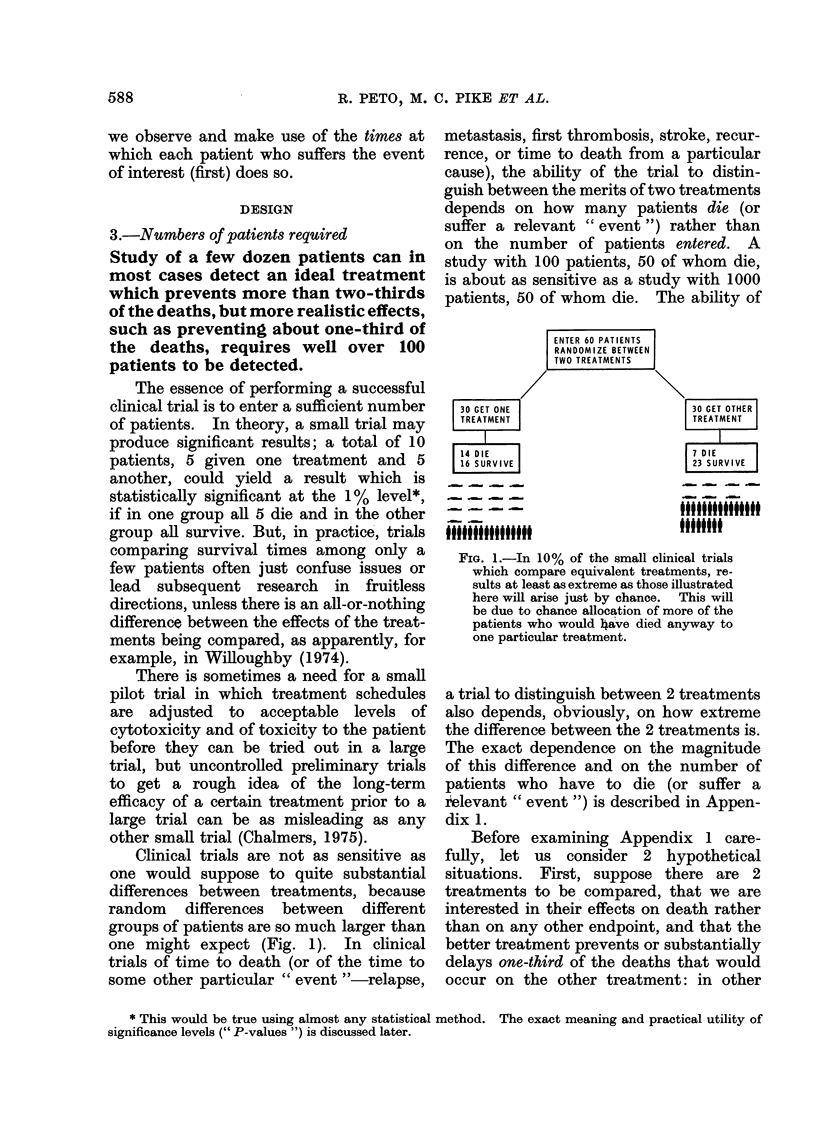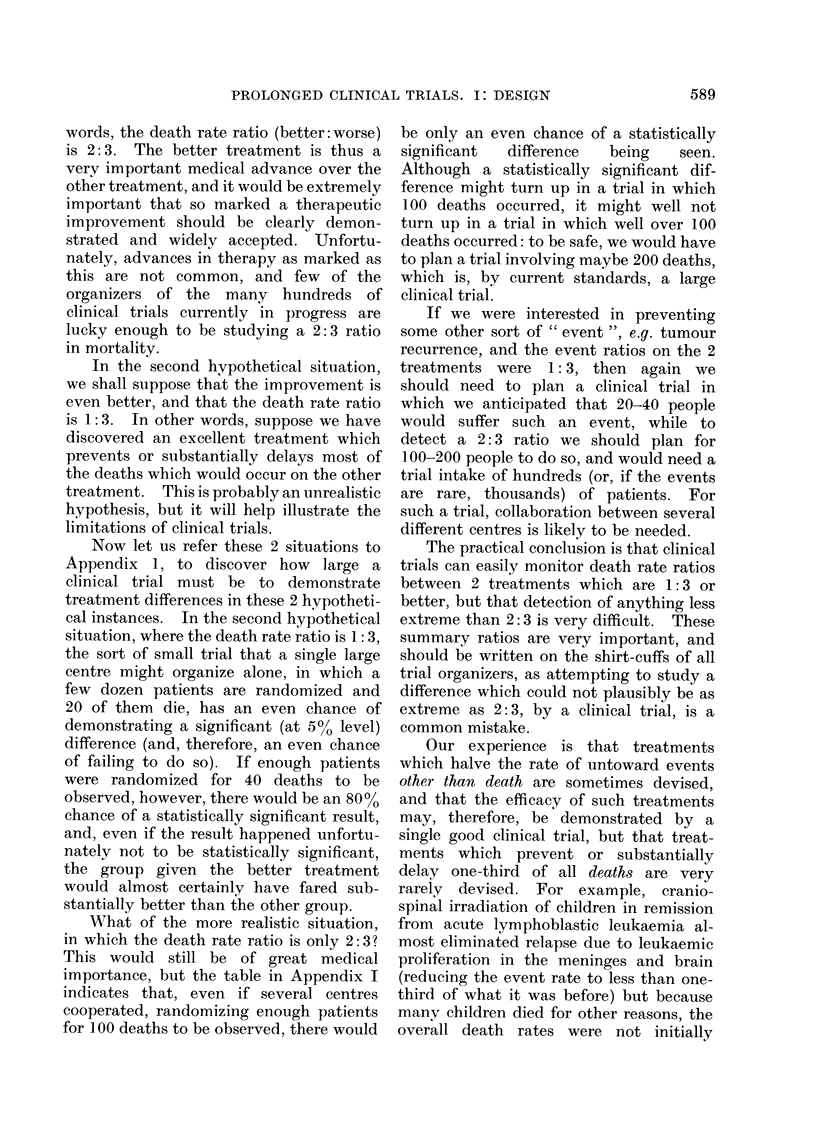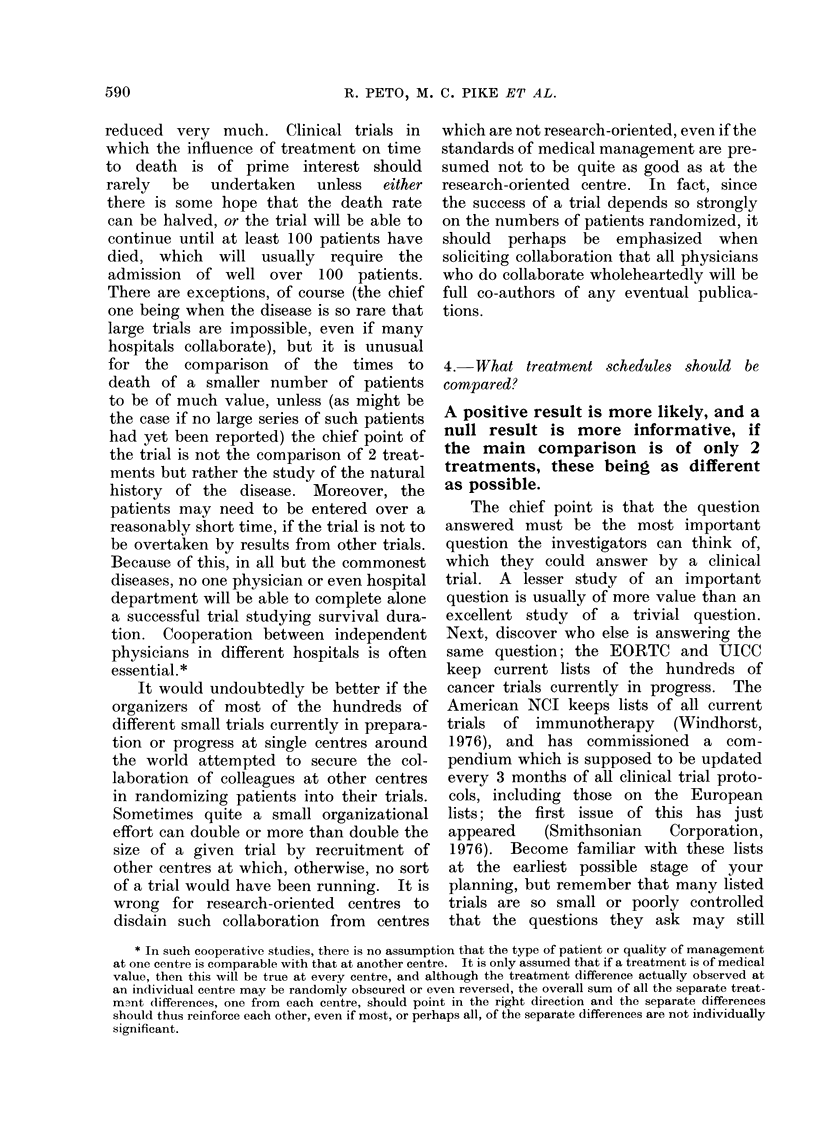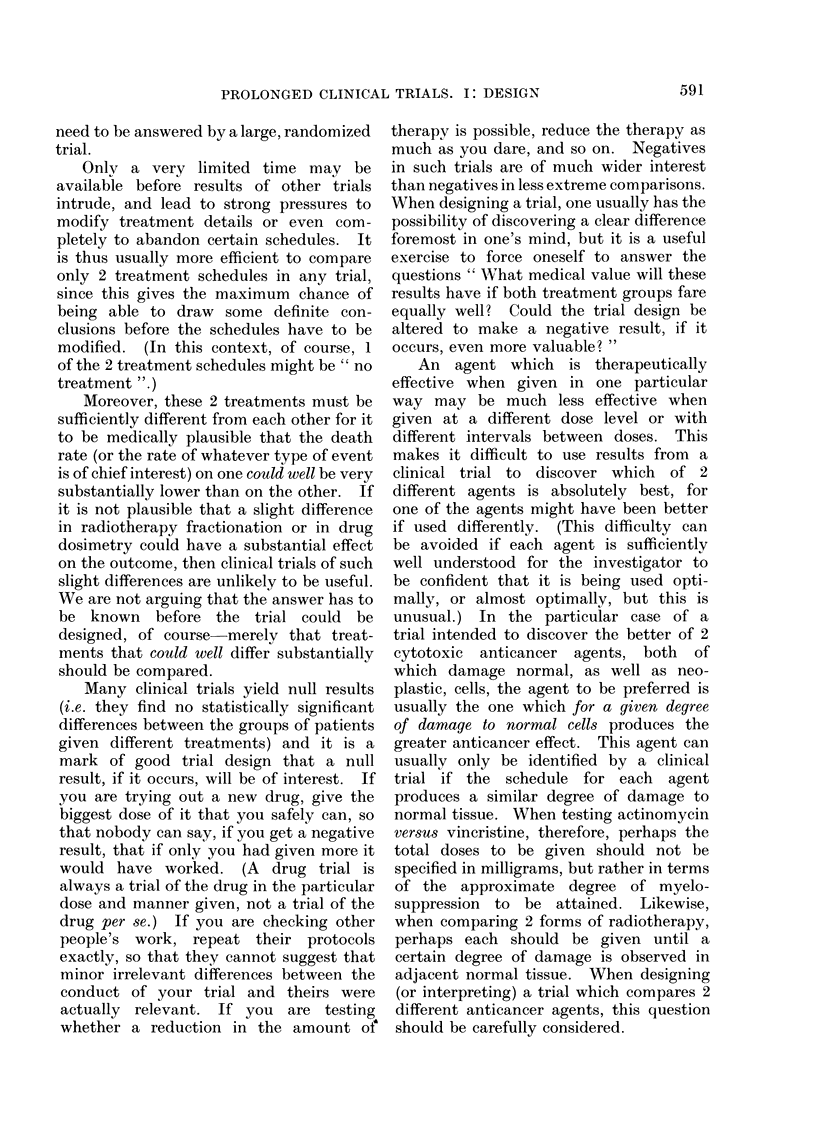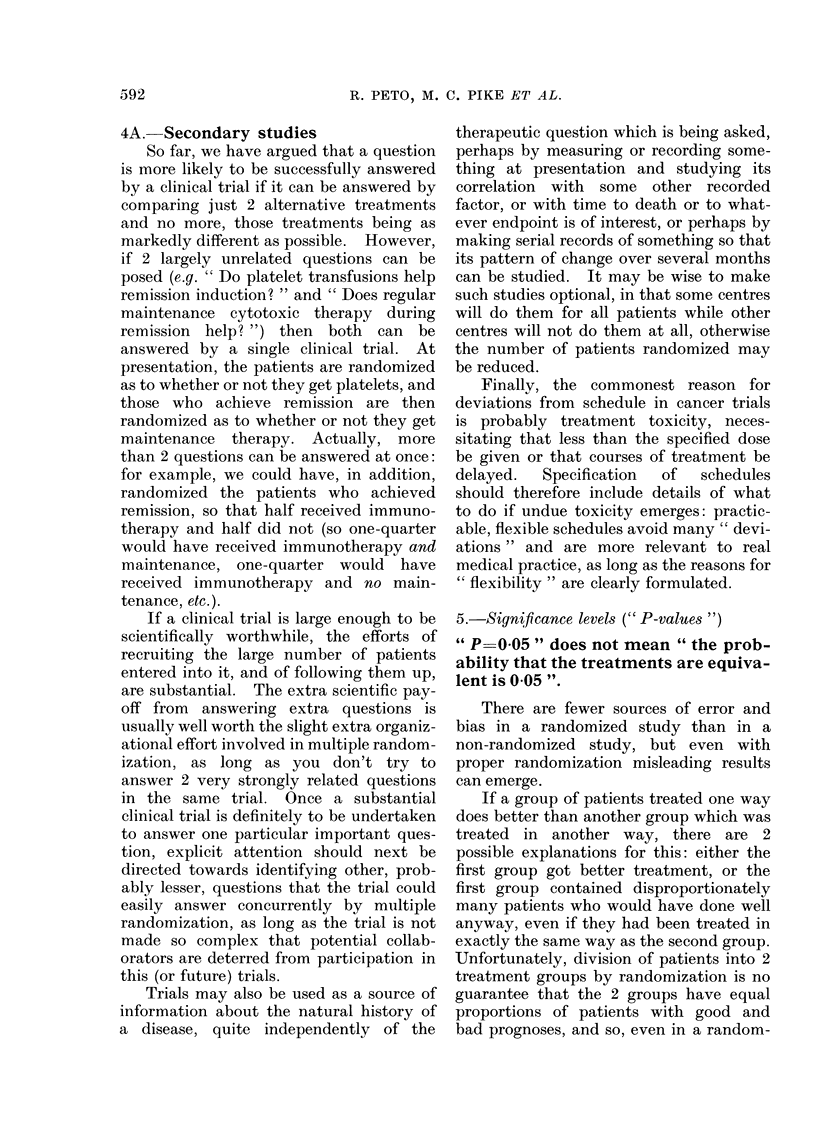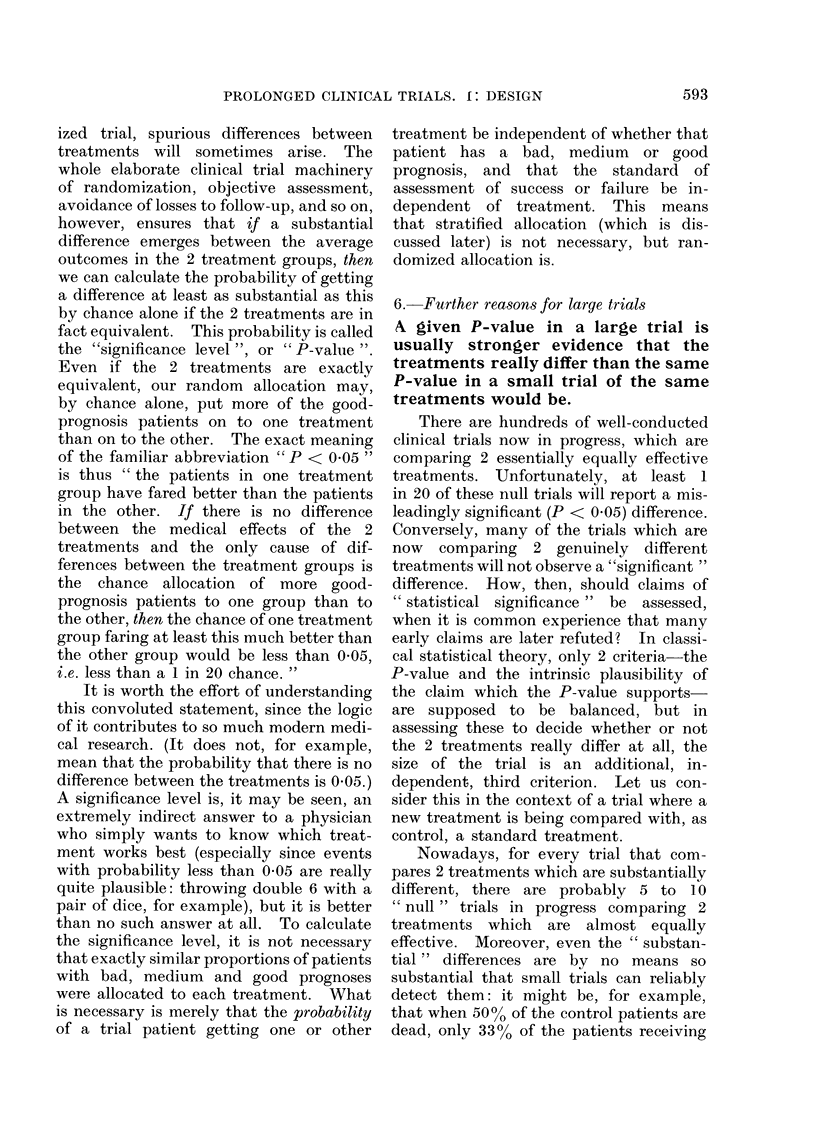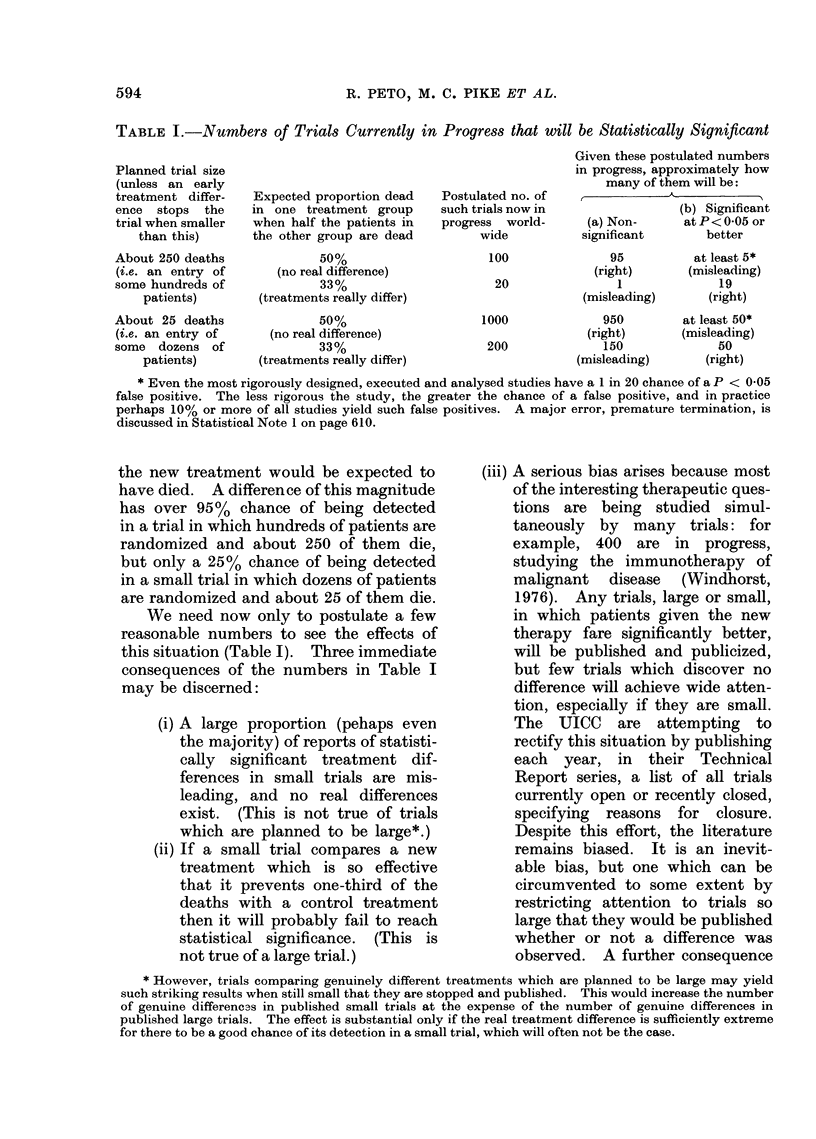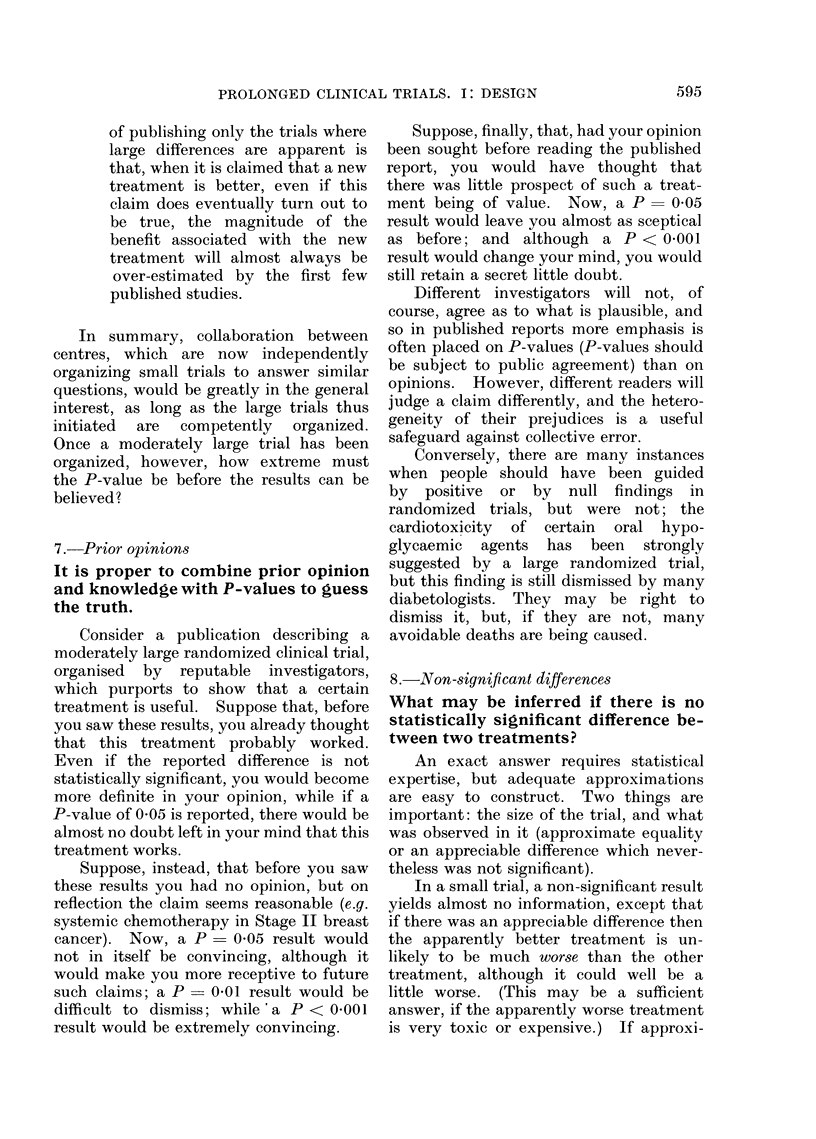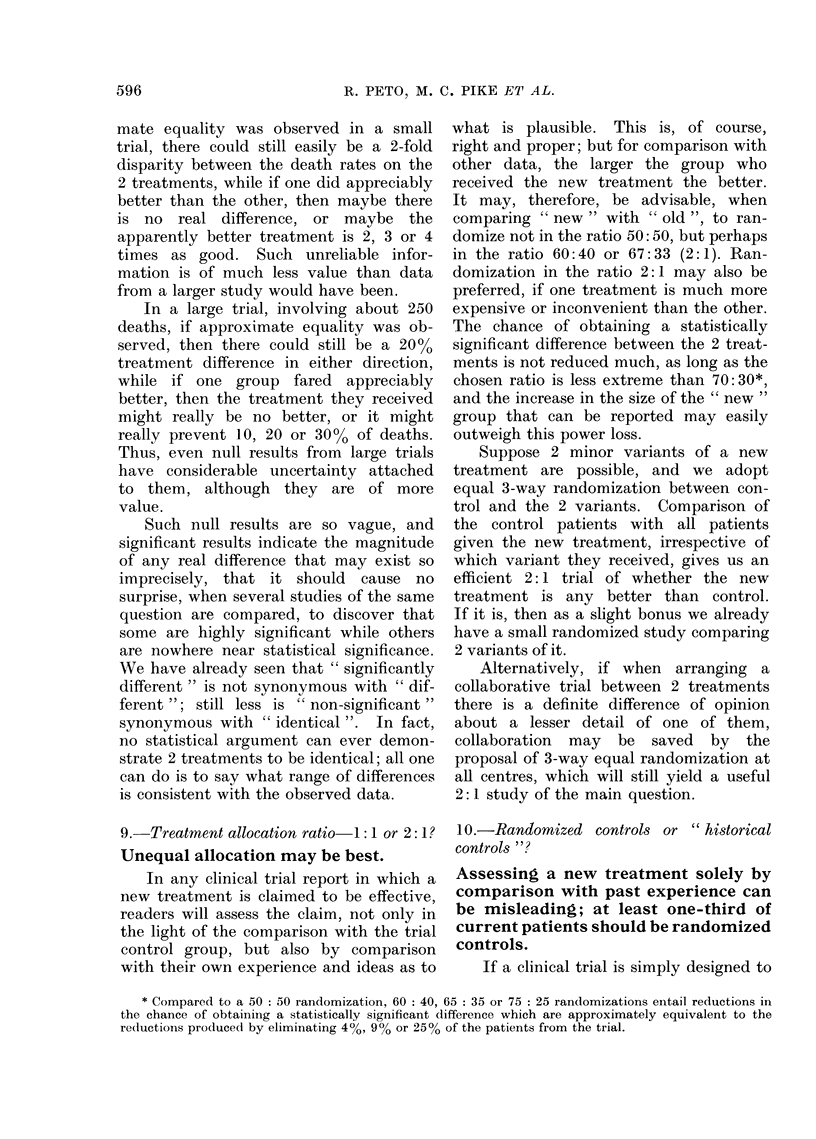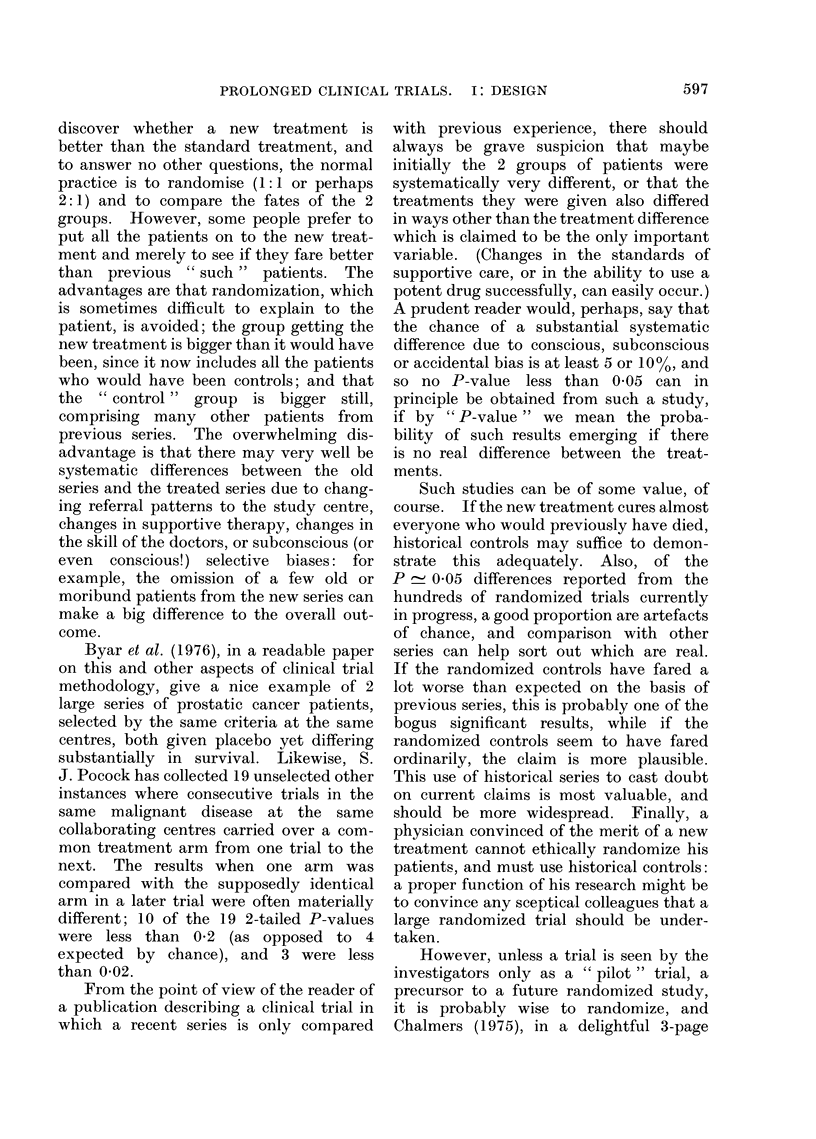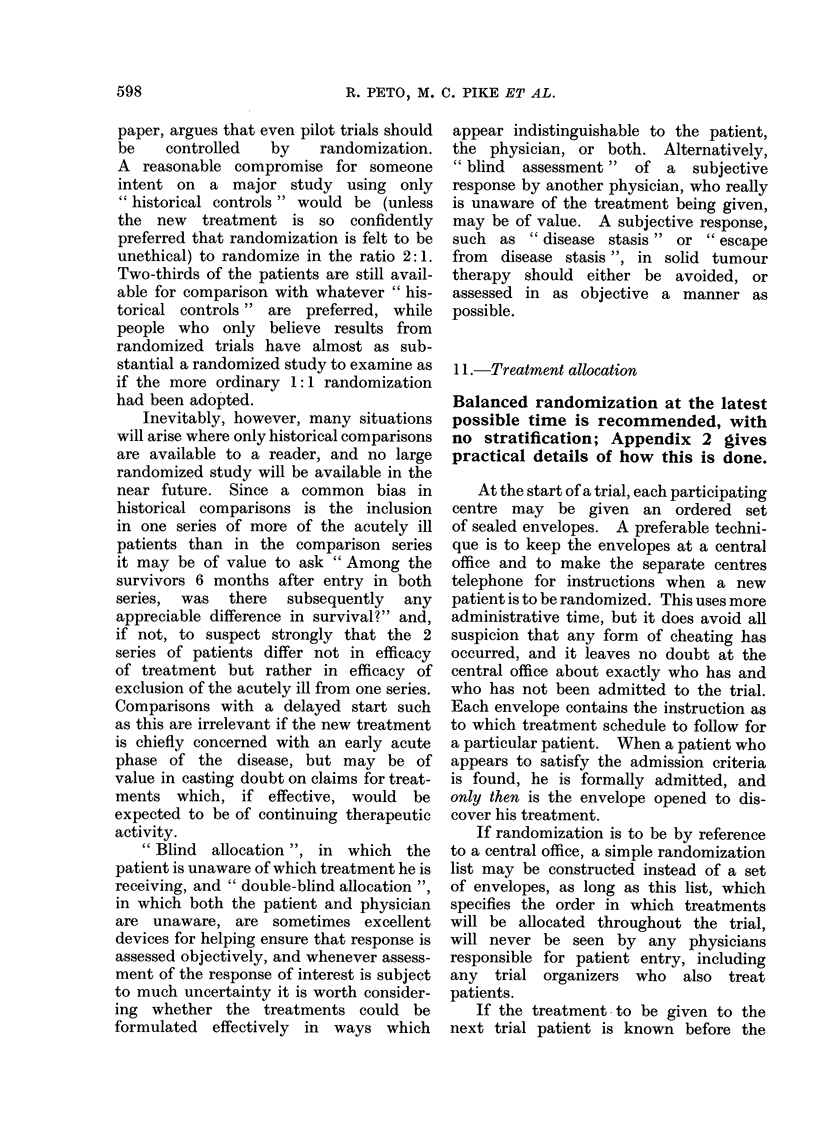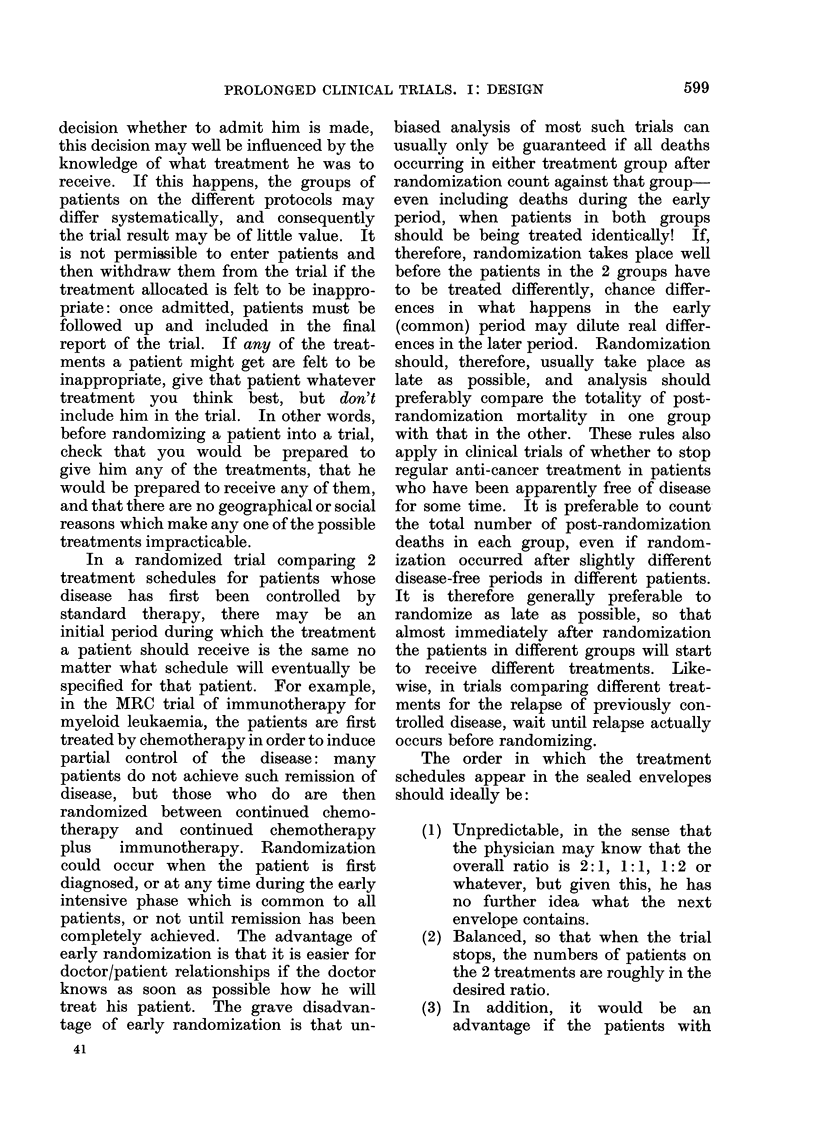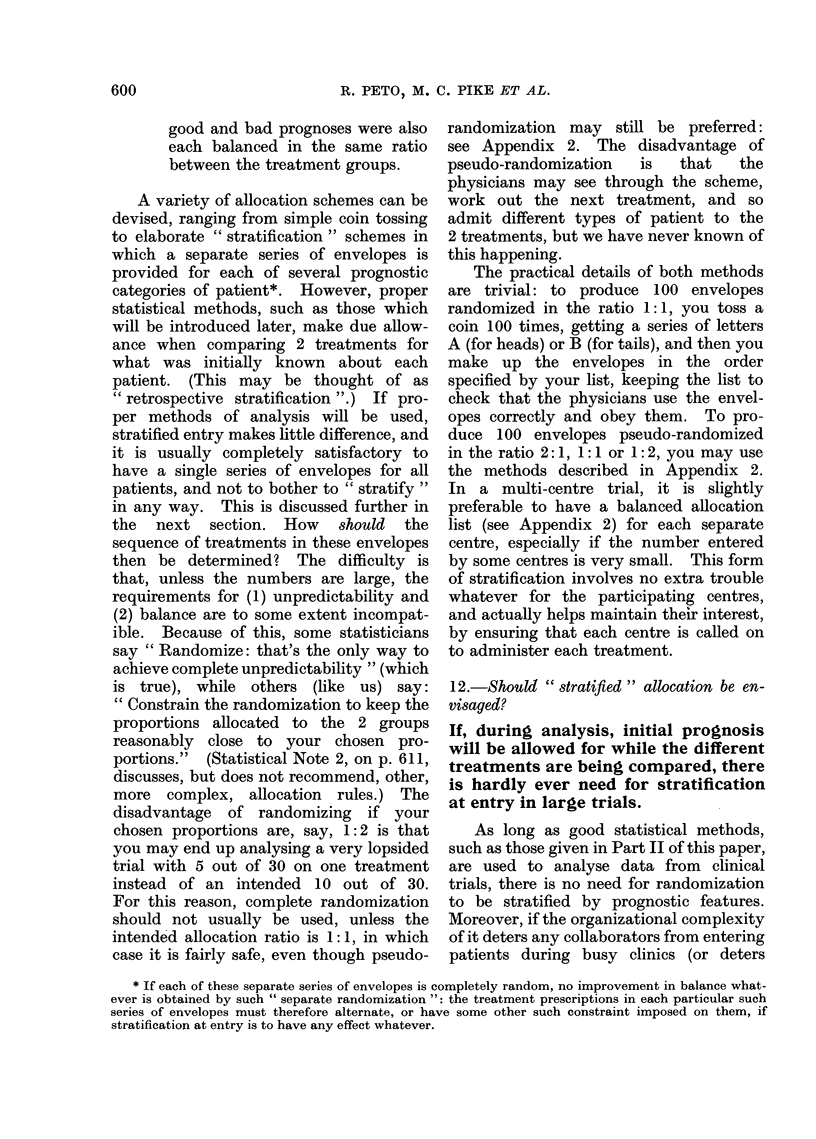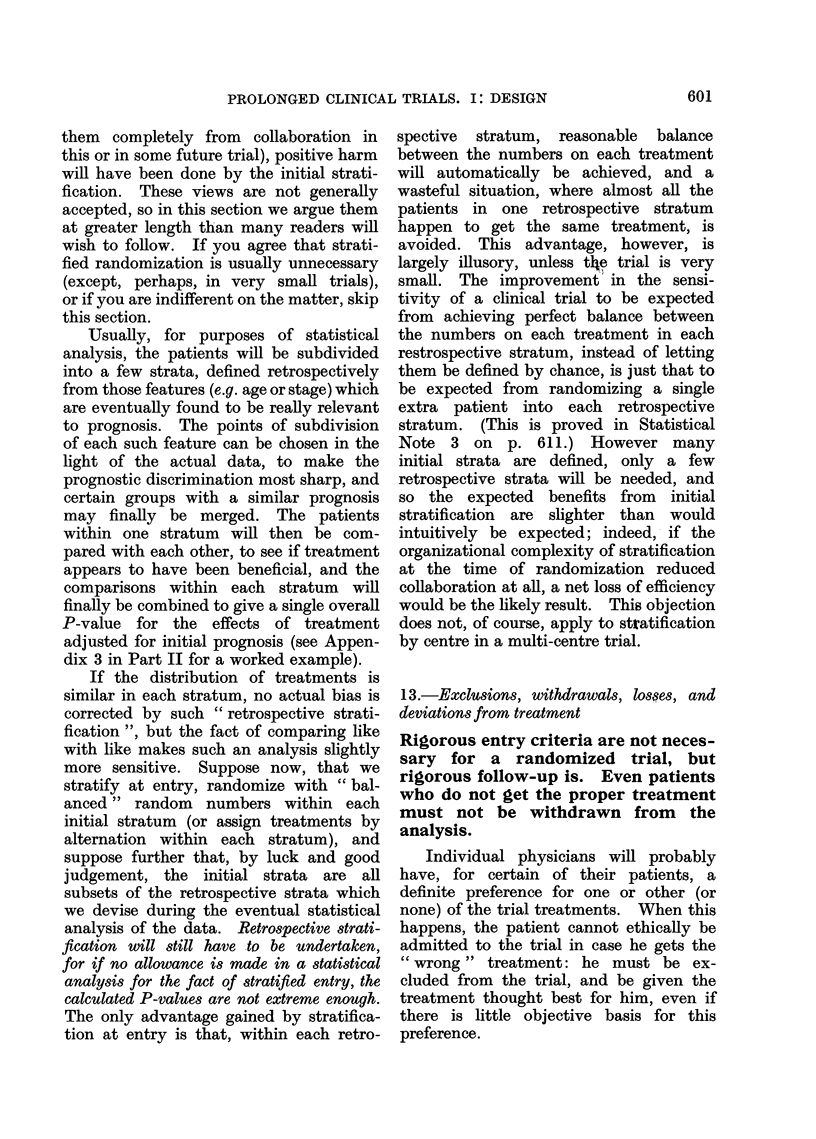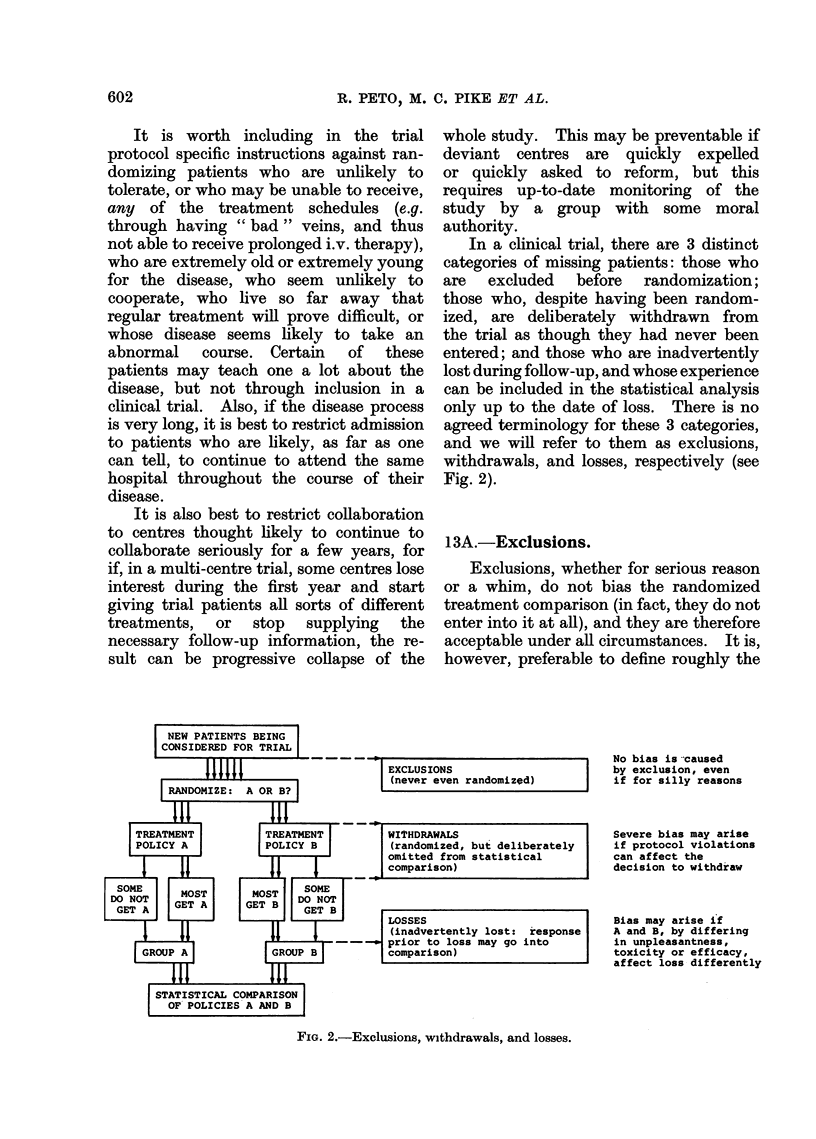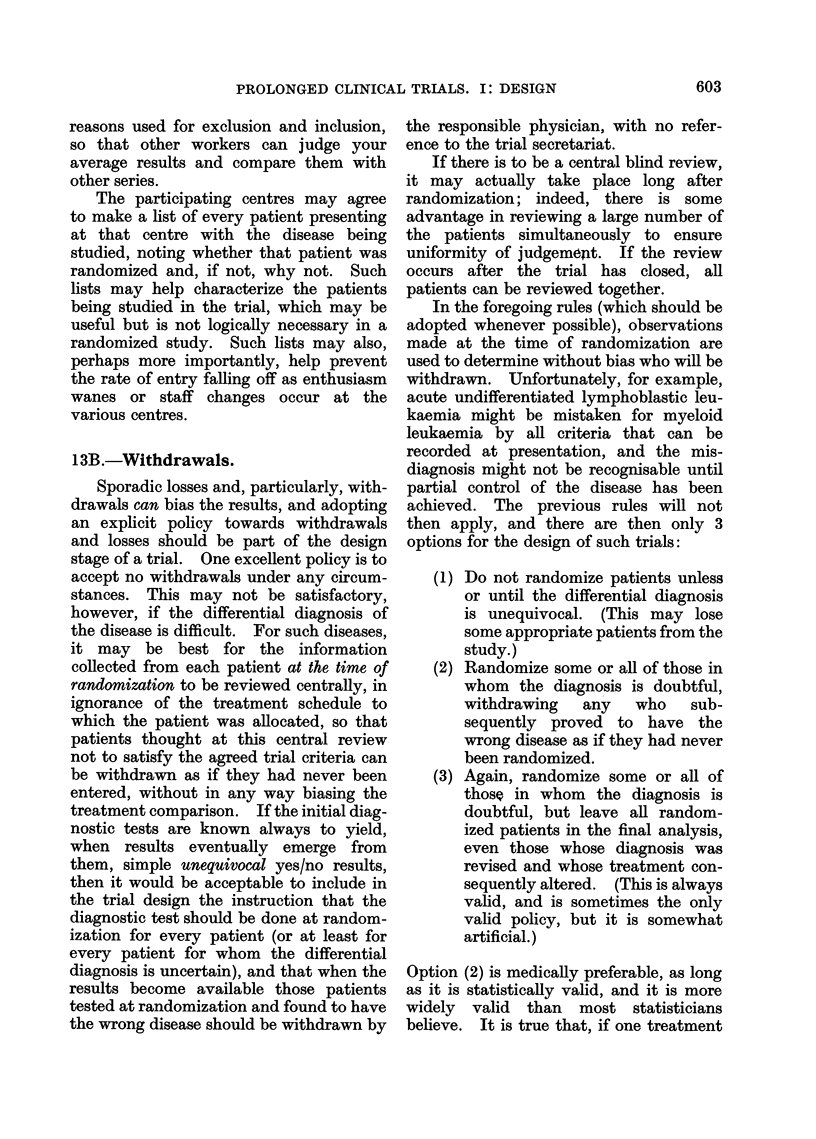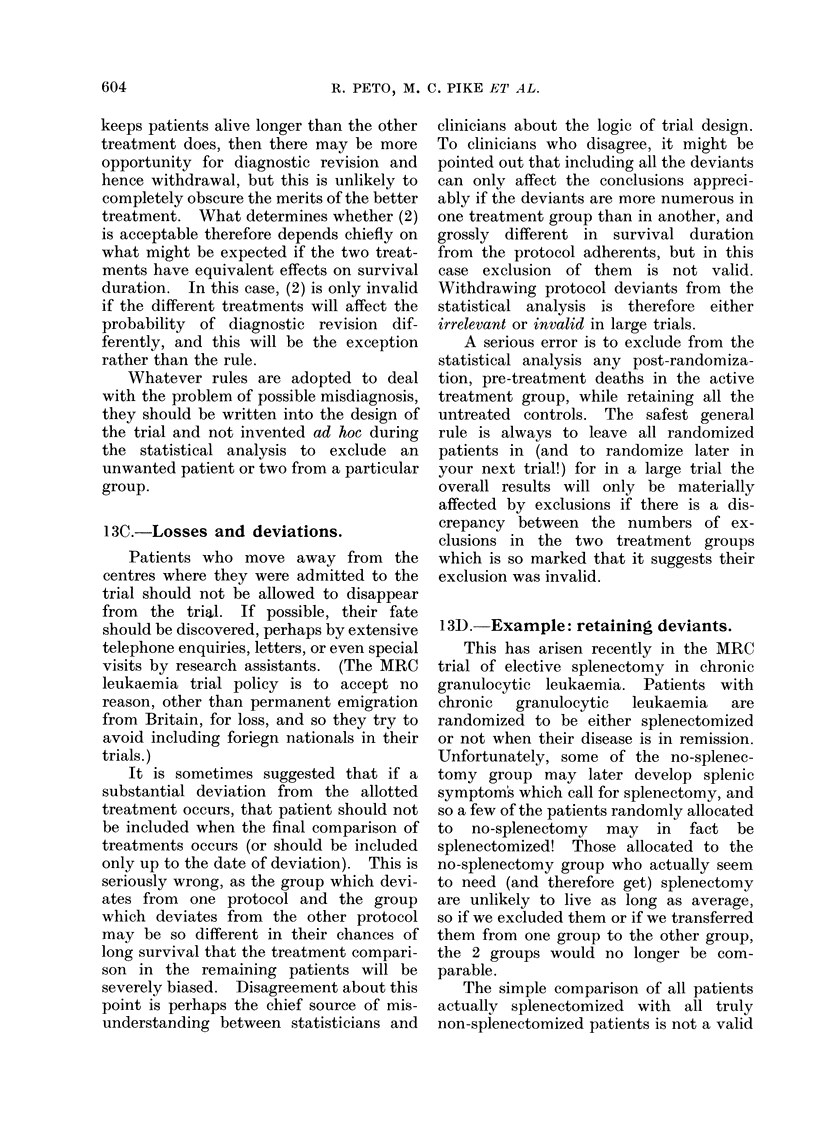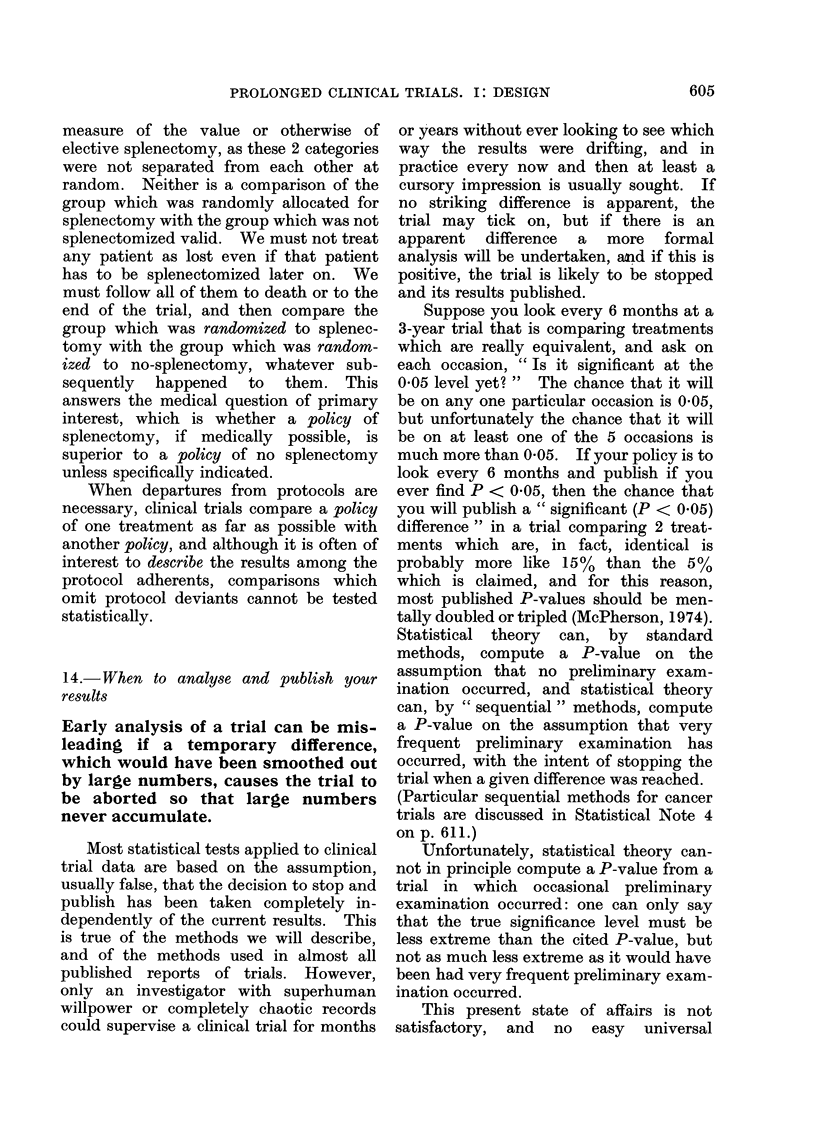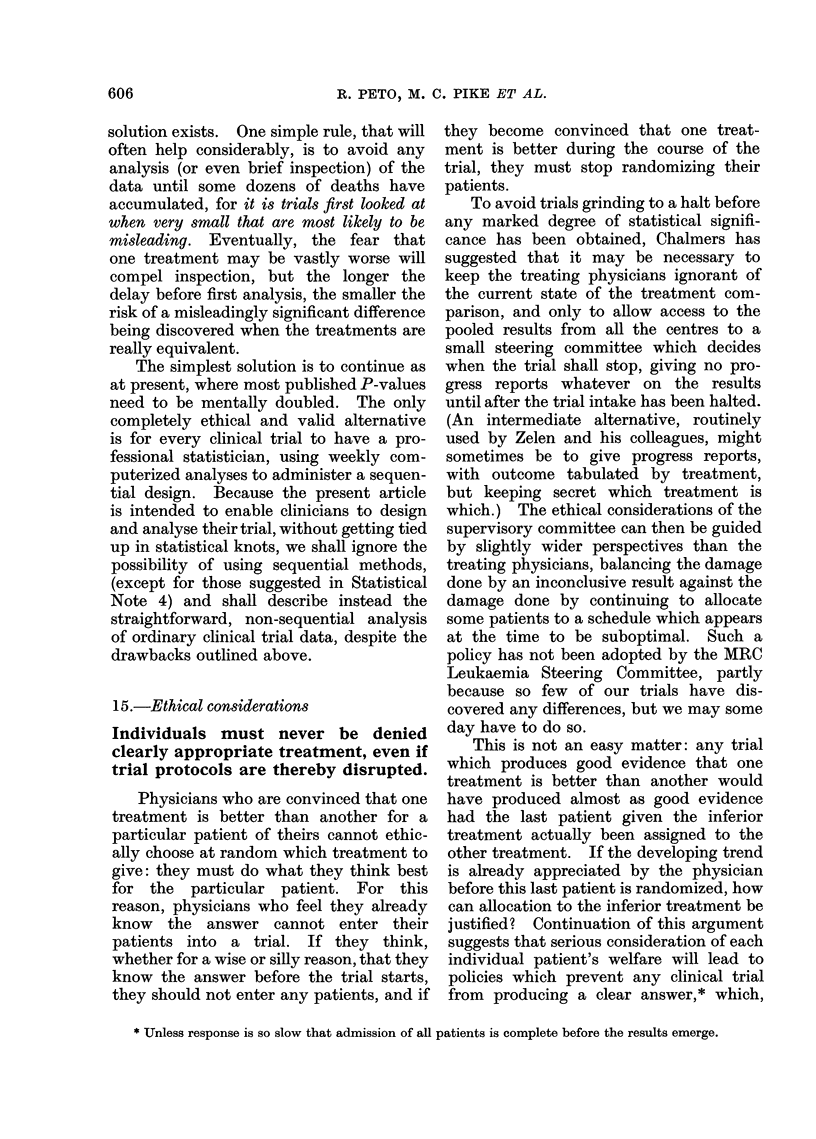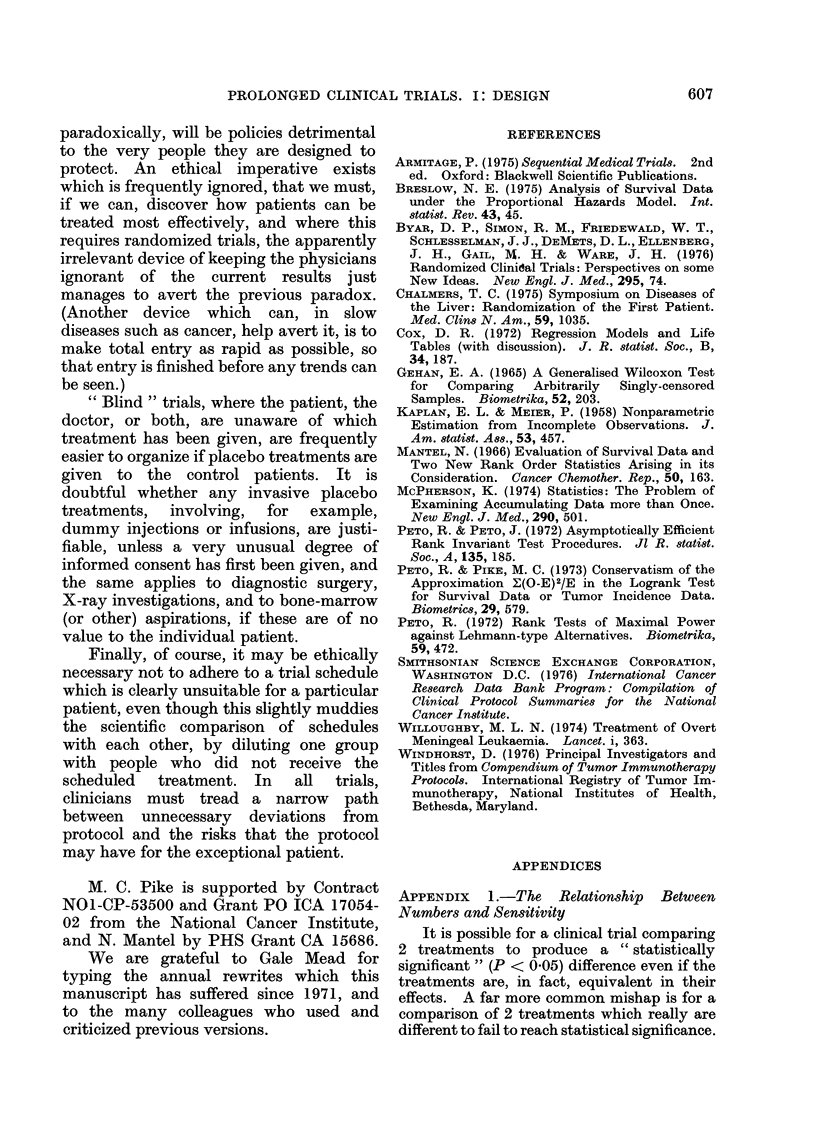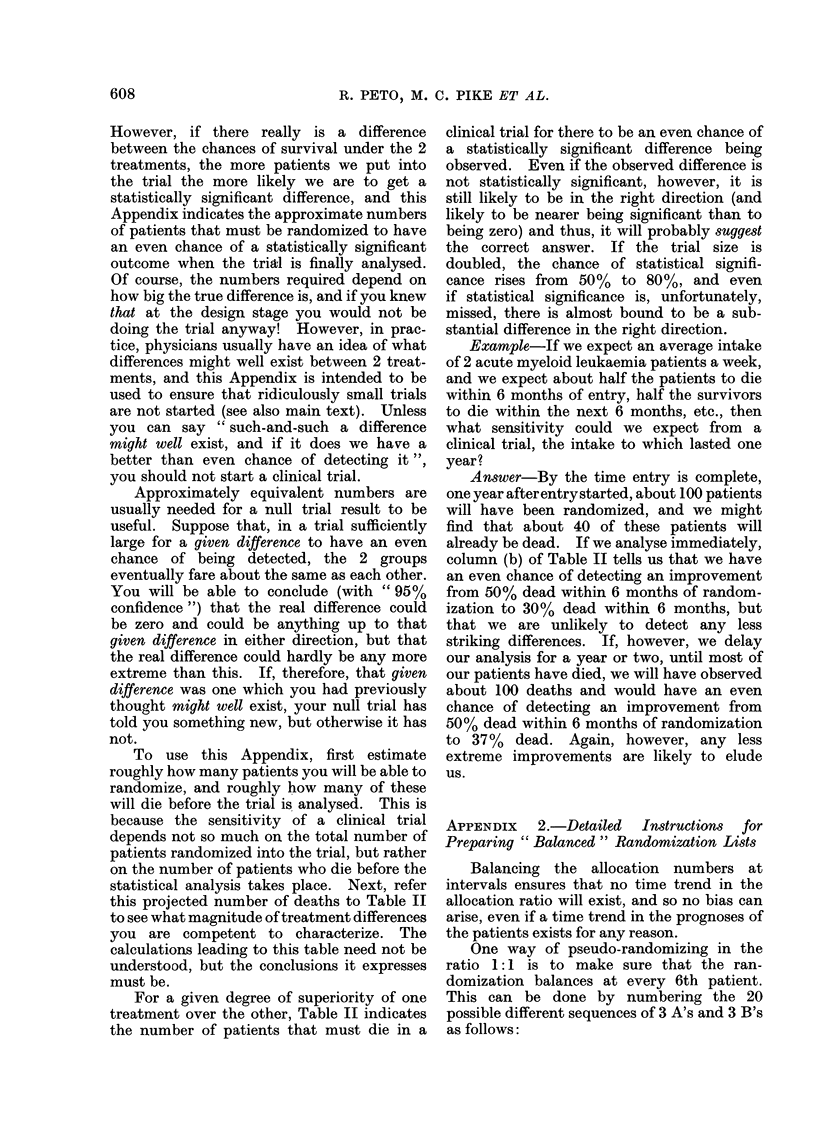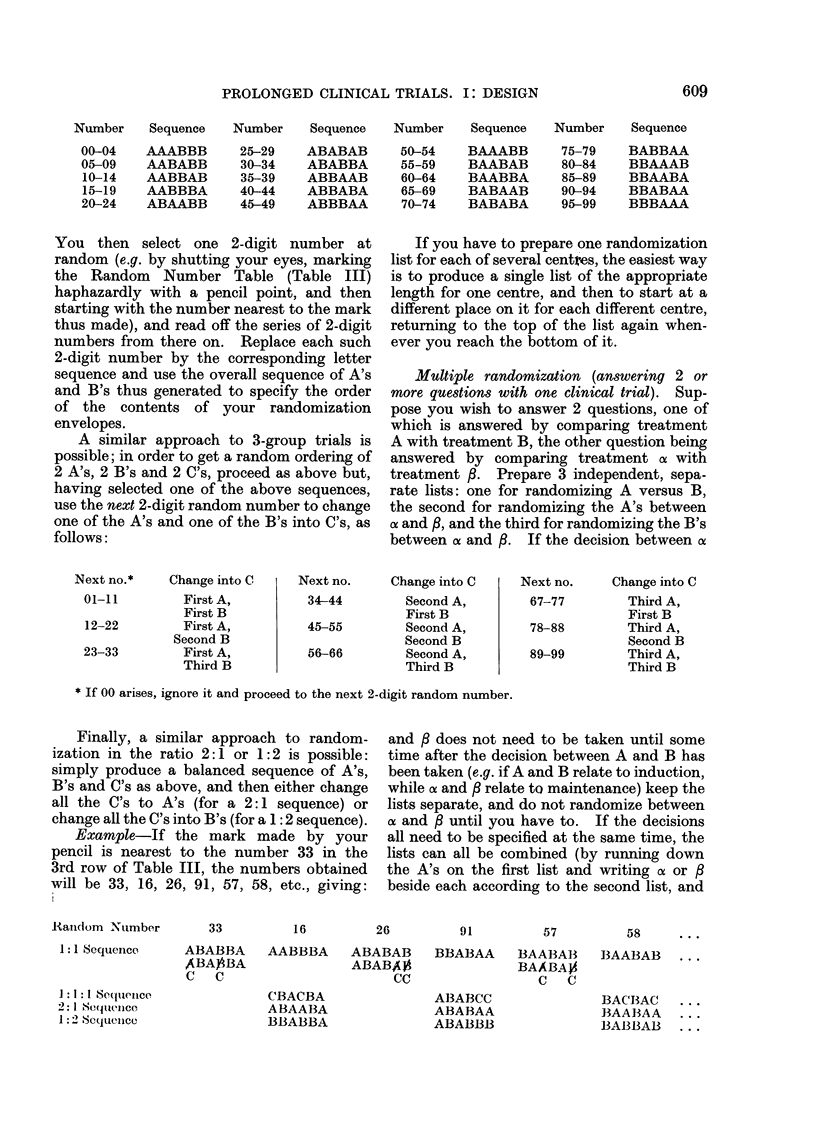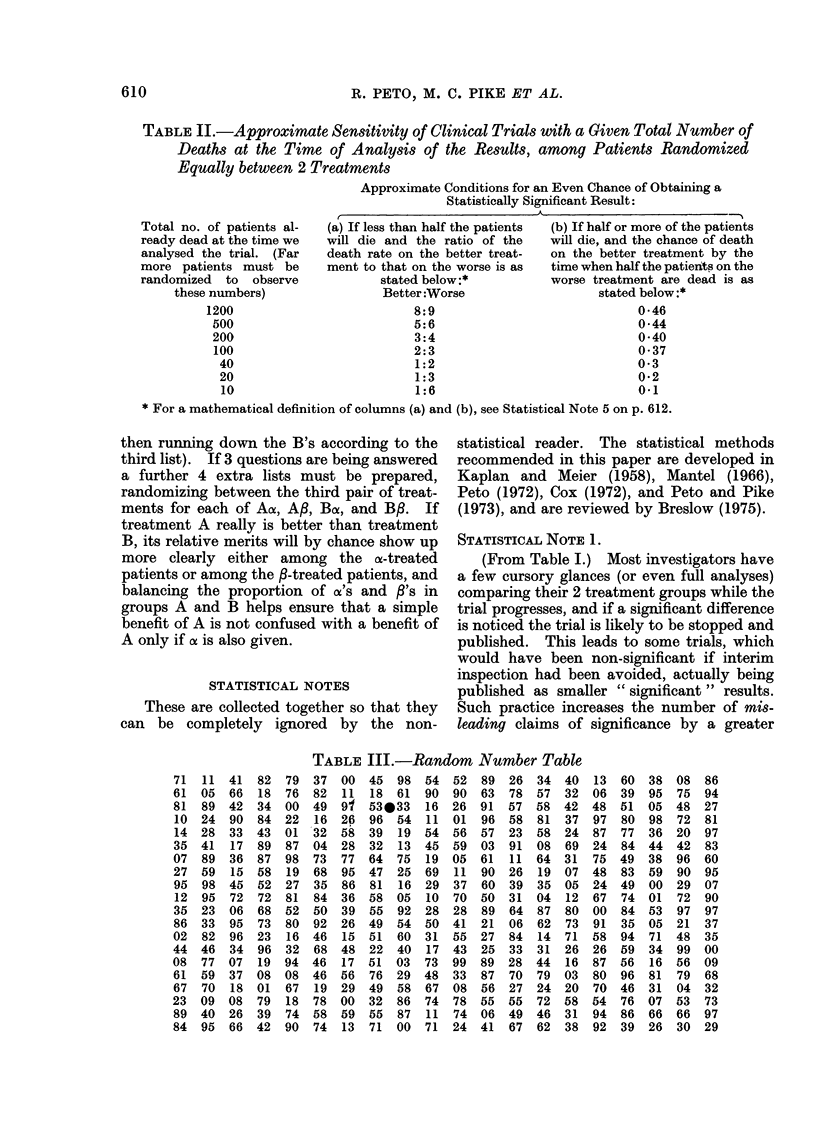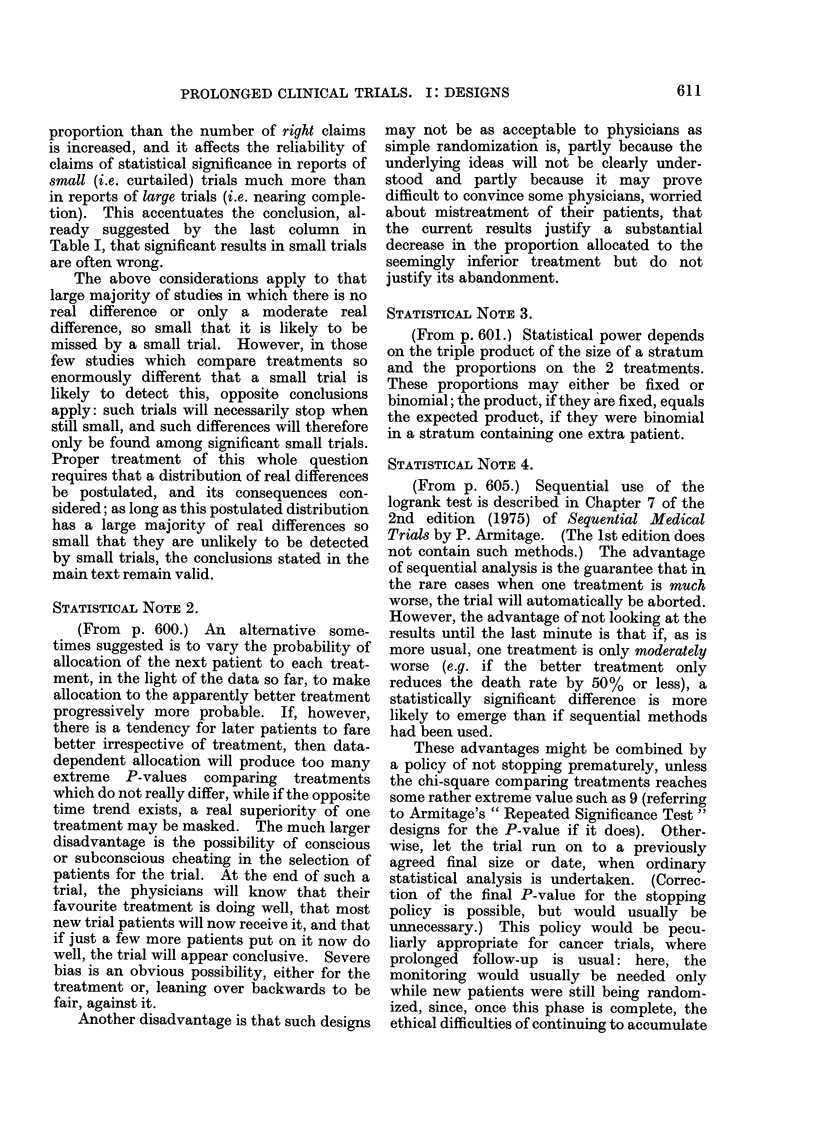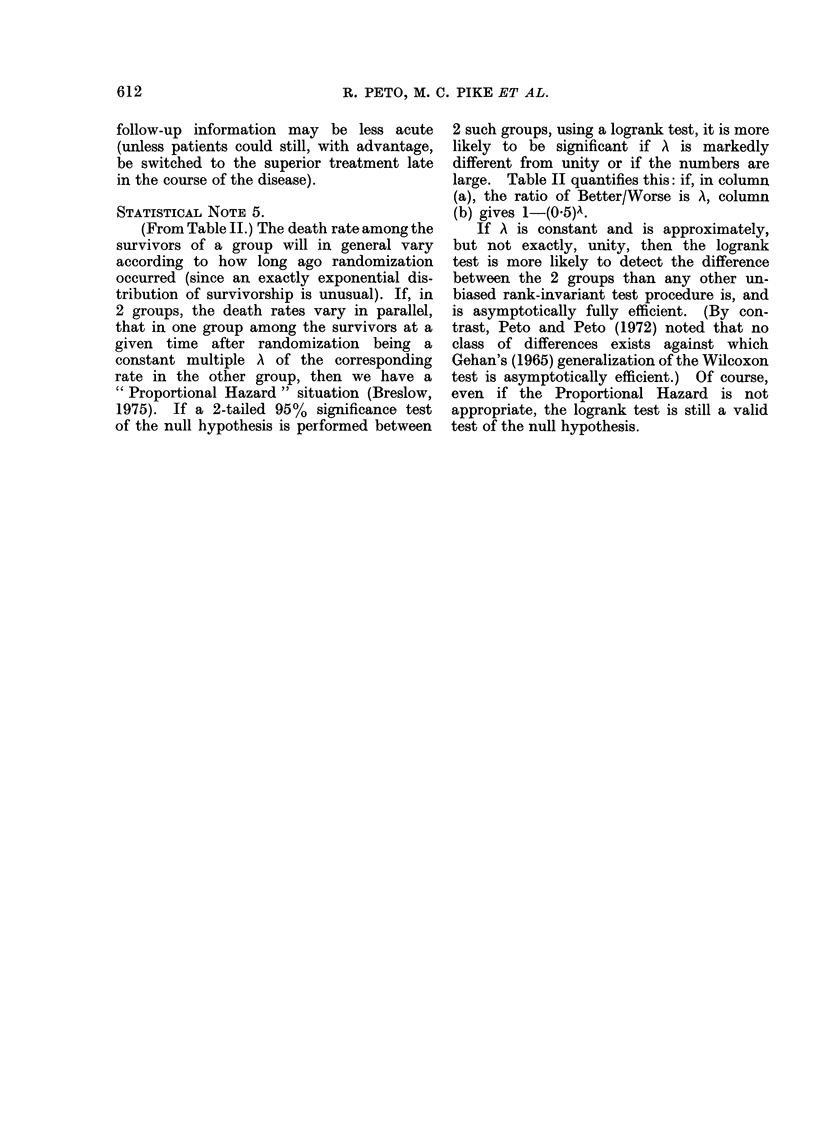# Design and analysis of randomized clinical trials requiring prolonged observation of each patient. I. Introduction and design.

**DOI:** 10.1038/bjc.1976.220

**Published:** 1976-12

**Authors:** R. Peto, M. C. Pike, P. Armitage, N. E. Breslow, D. R. Cox, S. V. Howard, N. Mantel, K. McPherson, J. Peto, P. G. Smith

## Abstract

The Medical Research Council has for some years encouraged collaborative clinical trials in leukaemia and other cancers, reporting the results in the medical literature. One unreported result which deserves such publication is the development of the expertise to design and analyse such trials. This report was prepared by a group of British and American statisticians, but it is intended for people without any statistical expertise. Part I, which appears in this issue, discusses the design of such trials; Part II, which will appear separately in the January 1977 issue of the Journal, gives full instructions for the statistical analysis of such trials by means of life tables and the logrank test, including a worked example, and discusses the interpretation of trial results, including brief reports of 2 particular trials. Both parts of this report are relevant to all clinical trials which study time to death, and wound be equally relevant to clinical trials which study time to other particular classes of untoward event: first stroke, perhaps, or first relapse, metastasis, disease recurrence, thrombosis, transplant rejection, or death from a particular cause. Part I, in this issue, collects together ideas that have mostly already appeared in the medical literature, but Part II, next month, is the first simple account yet published for non-statistical physicians of how to analyse efficiently data from clinical trials of survival duration. Such trials include the majority of all clinical trials of cancer therapy; in cancer trials,however, it may be preferable to use these statistical methods to study time to local recurrence of tumour, or to study time to detectable metastatic spread, in addition to studying total survival. Solid tumours can be staged at diagnosis; if this, or any other available information in some other disease is an important determinant of outcome, it can be used to make the overall logrank test for the whole heterogeneous trial population more sensitive, and more intuitively satisfactory, for it will then only be necessary to compare like with like, and not, by chance, Stage I with Stage III.


*Br. J. Cancer* (1976) **34**, 585.

### DESIGN AND ANALYSIS OF RANDOMIZED CLINICAL TRIALS REQUIRING PROLONGED OBSERVATION OF EACH PATIENT

### I. INTRODUCTION AND DESIGN

R. PETO,[1] M. C. PIKE,[2] P. ARMITAGE,[1] N. E. BRESLOW,[3] D. R. COX,[4] S. V. HOWARD,[5] N. MANTEL,[6] K. McPHERSON,[1] J. PETO[1] AND P. G. SMITH[1]

*From [1]Oxford University, [2]University of Southern California, [3]University of Seattle, [4]Imperial College, London, [5]U.C.H. Medical School and [6]George Washington University*

*Report to the Medical Research Council's Leukaemia Steering Committee; Chairman, Professor Sir Richard Doll*

Received 22 December 1975 Accepted 25 August 1976

**Summary.—The Medical Research Council has for some years encouraged collaborative clinical trials in leukaemia and other cancers, reporting the results in the medical literature. One unreported result which deserves such publication is the development of the expertise to design and analyse such trials. This report was prepared by a group of British and American statisticians, but it is intended for people without any statistical expertise. Part I, which appears in this issue, discusses the design of such trials; Part II, which will appear separately in the January 1977 issue of the Journal, gives full instructions for the statistical analysis of such trials by means of life tables and the logrank test, including a worked example, and discusses the interpretation of trial results, including brief reports of 2 particular trials.**

**Both parts of this report are relevant to all clinical trials which study time to death, and would be equally relevant to clinical trials which study time to other particular classes of untoward event: first stroke, perhaps, or first relapse, metastasis, disease recurrence, thrombosis, transplant rejection, or death from a particular cause. Part I, in this issue, collects together ideas that have mostly already appeared in the medical literature, but Part II, next month, is the first simple account yet published for non-statistical physicians of how to analyse efficiently data from clinical trials of survival duration. Such trials include the majority of all clinical trials of cancer therapy; in cancer trials, however, it may be preferable to use these statistical methods to study time to local recurrence of tumour, or to study time to detectable metastatic spread, in addition to studying total survival. Solid tumours can be staged at diagnosis; if this, or any other available information in some other disease is an important determinant of outcome, it can be used to make the overall logrank test for the whole heterogeneous trial population more sensitive, and more intuitively satisfactory, for it will then only be necessary to compare like with like, and not, by chance, Stage I with Stage III.**

CONTENTS

Many of these sections may be read out of context, or skipped while reading the main report. The sections on Analysis (Part II), which describe how to draw life tables and to compute $P$-values by the logrank test, can be used in isolation from the earlier sections on Design (Part I).

Requests for reprints to: R. Peto, Radcliffe Infirmary, Oxford, England; or to: M. C. Pike, University of Southern California School of Medicine, Los Angeles, California 90033, U.S.A. Reprints of both parts will be sent to those who request reprints of either part.

INTRODUCTION PAGE

1.—General aims . . . . . . . . . . . . . 586

2.—Event times . . . . . . . . . . . . . 587

DESIGN

3.—Numbers of patients required . . . . . . . . . . 588

4.—What treatment schedules should be compared? . . . . . . 590

5.—Significance levels (" $P$-values ") . . . . . . . . . 592

6.—Further reasons for large trials . . . . . . . . . . 593

7.—Prior opinions. . . . . . . . . . . . . 595

8.—Non-significant differences . . . . . . . . . . 595

9.—Treatment allocation ratio: 1:1 or 2:1? . . . . . . . . 596

10.—Randomized controls or " historical controls "? . . . . . . 596

11.—Treatment allocation . . . . . . . . . . . 598

12.—Should " stratified " allocation be envisaged? . . . . . . 600

13.—Exclusions, withdrawals, losses and deviations from treatment . . 601

14.—When to analyse and publish your results . . . . . . . 605

15.—Ethical considerations . . . . . . . . . . . 606

REFERENCES FOR PART I . . . . . . . . . . . . 607

APPENDICES FOR PART I
1.—The relationship between numbers and sensitivity . . . . . 607
2.—Detailed instructions for preparing " balanced " randomization lists . 608

STATISTICAL NOTES FOR PART I . . . . . . . . . . . 610

(Part II. Analysis and Examples will appear in Vol. 35, No. 1, January 1977)

### INTRODUCTION

### 1.—*General aims*

Many clinical trials compare survival duration among cancer patients randomly allocated to different treatments. There has been much investigation in the statistical literature of possible ways of interpreting the data from such trials, the surprising outcome of which has been the discovery that two techniques (life table graphs and logrank $P$-values), which are so simple that they are easily mastered by non-statisticians, are commonly more accurate and more sensitive than any of the elaborate alternatives that have been considered. Part II of this report, which will appear in the next issue, describes these two techniques in sufficient detail for them to be performed entirely without statistical guidance. Part I, in this issue, explains, without using specialized statistical language, statistical ideas about numbers of patients, withdrawals, stratification and so on. So that no reference to any other papers or tables should be necessary, even such commonplace statistical things as chi-square (in Part II) and random number tables are supplied and explained.

This report addresses itself to the more quantifiable aspects of clinical trials—treatment comparisons, treatment toxicities, treatment effects in special subgroups, recognition of prognostic features, and so on—while ignoring the important indirect benefits of clinical trials. For example, there is variation in clinical practice between different hospitals, and in a collaborative trial this is likely to be discussed, leading perhaps to a valuable exchange of ideas and to some improvements. Even if this does not occur, the imposition of a treatment schedule actually confers a freedom from having to devise separately each detail of treatment for each different patient, which may make patient management easier and, perhaps, more successful. Another indirect benefit is that a pattern may be noticed in the data which, even if it could well be due just to chance, suggests a line of thought which proves fruitful in this, or some future, study.

It would be unfortunate if our later emphasis on definite answers and large trials were to dissuade prospective organizers from planning a small clinical trial in a hitherto little-studied condition if the alternative is no trial at all. However, most small trials could, with profit, be larger if the organizers attempted collaboration with other hospitals.

The detail needed to explain a concept such as the $P$-value to readers who are not clear about its true meaning and usefulness would be tiresome to readers who are, and so both parts of the report are divided into sections, each preceded by a sentence summarizing it, so that the whole thing can be looked through without wasting time reading sections which are already understood: likewise, because of the contents list and section summaries, particular details (*e.g.* arithmetic techniques) should be easy to refer to, if only these are wanted. The report may help physicians to dispense with specialized statistical advice on some occasions, and make more critical use of it on others. It should also be of interest to statisticians who have not yet specialized in such data. For statistical readers, a series of " statistical notes " is referred to throughout the text. These are collected together at the end so as not to distract the general reader from the main text, which is self-sufficient without them.

The collective noun for statisticians is said to be " A variance of statisticians ", and so, although the whole report is largely consensual, some of us may differ from it in certain particulars, as may some members of the committee to which it is addressed.

2.—*Event times*

**Whatever index of failure is of interest, one should not only count how many people " fail " but also see *when* they failed.**

A common form of clinical trial compares treatments which are intended to prevent or delay death from a particular disease. If the course of the disease is very rapid (*e.g.* acute liver failure) and it is unimportant whether a dying patient lives a few days longer or not, a count of the numbers of deaths and survivors on each treatment is all that is required. However, if (as with most forms of neoplastic disease) an appreciable proportion of the patients do die of the disease, but death may take some considerable time, it is possible to achieve a more sensitive assessment of the value of each treatment by looking not only at how many patients died, but also at how long after entry they died. The best way of doing this is quite simple, but it is easy to overlook when first confronted with the problem. This also applies to any clinical trial which is concerned with the prevention of some other untoward " event " (or " endpoint ") that may eventually affect some or all of the patients. If these events may not occur for some time after starting treatment, it is worth looking at the times at which the events occur as well as counting the number of events in each group of patients. The sort of untoward events which could be studied in such a clinical trial might be myocardial infarctions, leukaemia relapses, strokes, metastatic developments, death from one of a certain set of specified causes (ignoring deaths from other causes), transplant rejection episodes and so on. In each of these studies, it is usually possible to do a more informative analysis than a simple tabulation of the numbers of patients on each treatment who suffered the event of interest. Whether this event is death or local solid tumour recurrence or something else, the design principles and statistical methods are virtually the same:

we observe and make use of the *times* at which each patient who suffers the event of interest (first) does so.

### DESIGN

### 3.—*Numbers of patients required*

**Study of a few dozen patients can in most cases detect an ideal treatment which prevents more than two-thirds of the deaths, but more realistic effects, such as preventing about one-third of the deaths, requires well over 100 patients to be detected.**

The essence of performing a successful clinical trial is to enter a sufficient number of patients. In theory, a small trial may produce significant results; a total of 10 patients, 5 given one treatment and 5 another, could yield a result which is statistically significant at the 1% level*, if in one group all 5 die and in the other group all survive. But, in practice, trials comparing survival times among only a few patients often just confuse issues or lead subsequent research in fruitless directions, unless there is an all-or-nothing difference between the effects of the treatments being compared, as apparently, for example, in Willoughby (1974).

There is sometimes a need for a small pilot trial in which treatment schedules are adjusted to acceptable levels of cytotoxicity and of toxicity to the patient before they can be tried out in a large trial, but uncontrolled preliminary trials to get a rough idea of the long-term efficacy of a certain treatment prior to a large trial can be as misleading as any other small trial (Chalmers, 1975).

Clinical trials are not as sensitive as one would suppose to quite substantial differences between treatments, because random differences between different groups of patients are so much larger than one might expect (Fig. 1). In clinical trials of time to death (or of the time to some other particular " event "—relapse, metastasis, first thrombosis, stroke, recurrence, or time to death from a particular cause), the ability of the trial to distinguish between the merits of two treatments depends on how many patients *die* (or suffer a relevant " event ") rather than on the number of patients *entered.* A study with 100 patients, 50 of whom die, is about as sensitive as a study with 1000 patients, 50 of whom die. The ability of

FIG. 1.—In 10% of the small clinical trials which compare equivalent treatments, results at least as extreme as those illustrated here will arise just by chance. This will be due to chance allocation of more of the patients who would have died anyway to one particular treatment.

a trial to distinguish between 2 treatments also depends, obviously, on how extreme the difference between the 2 treatments is. The exact dependence on the magnitude of this difference and on the number of patients who have to die (or suffer a relevant " event ") is described in Appendix 1.

Before examining Appendix 1 carefully, let us consider 2 hypothetical situations. First, suppose there are 2 treatments to be compared, that we are interested in their effects on death rather than on any other endpoint, and that the better treatment prevents or substantially delays *one-third* of the deaths that would occur on the other treatment: in other

* This would be true using almost any statistical method. The exact meaning and practical utility of significance levels (" *P*-values ") is discussed later.

words, the death rate ratio (better: worse) is 2:3. The better treatment is thus a very important medical advance over the other treatment, and it would be extremely important that so marked a therapeutic improvement should be clearly demonstrated and widely accepted. Unfortunately, advances in therapy as marked as this are not common, and few of the organizers of the many hundreds of clinical trials currently in progress are lucky enough to be studying a 2:3 ratio in mortality.

In the second hypothetical situation, we shall suppose that the improvement is even better, and that the death rate ratio is 1:3. In other words, suppose we have discovered an excellent treatment which prevents or substantially delays most of the deaths which would occur on the other treatment. This is probably an unrealistic hypothesis, but it will help illustrate the limitations of clinical trials.

Now let us refer these 2 situations to Appendix 1, to discover how large a clinical trial must be to demonstrate treatment differences in these 2 hypothetical instances. In the second hypothetical situation, where the death rate ratio is 1:3, the sort of small trial that a single large centre might organize alone, in which a few dozen patients are randomized and 20 of them die, has an even chance of demonstrating a significant (at 5% level) difference (and, therefore, an even chance of failing to do so). If enough patients were randomized for 40 deaths to be observed, however, there would be an 80% chance of a statistically significant result, and, even if the result happened unfortunately not to be statistically significant, the group given the better treatment would almost certainly have fared substantially better than the other group.

What of the more realistic situation, in which the death rate ratio is only 2:3? This would still be of great medical importance, but the table in Appendix I indicates that, even if several centres cooperated, randomizing enough patients for 100 deaths to be observed, there would be only an even chance of a statistically significant difference being seen. Although a statistically significant difference might turn up in a trial in which 100 deaths occurred, it might well not turn up in a trial in which well over 100 deaths occurred: to be safe, we would have to plan a trial involving maybe 200 deaths, which is, by current standards, a large clinical trial.

If we were interested in preventing some other sort of " event ", *e.g.* tumour recurrence, and the event ratios on the 2 treatments were 1:3, then again we should need to plan a clinical trial in which we anticipated that 20–40 people would suffer such an event, while to detect a 2:3 ratio we should plan for 100–200 people to do so, and would need a trial intake of hundreds (or, if the events are rare, thousands) of patients. For such a trial, collaboration between several different centres is likely to be needed.

The practical conclusion is that clinical trials can easily monitor death rate ratios between 2 treatments which are 1:3 or better, but that detection of anything less extreme than 2:3 is very difficult. These summary ratios are very important, and should be written on the shirt-cuffs of all trial organizers, as attempting to study a difference which could not plausibly be as extreme as 2:3, by a clinical trial, is a common mistake.

Our experience is that treatments which halve the rate of untoward events *other than death* are sometimes devised, and that the efficacy of such treatments may, therefore, be demonstrated by a single good clinical trial, but that treatments which prevent or substantially delay one-third of all *deaths* are very rarely devised. For example, cranio-spinal irradiation of children in remission from acute lymphoblastic leukaemia almost eliminated relapse due to leukaemic proliferation in the meninges and brain (reducing the event rate to less than one-third of what it was before) but because many children died for other reasons, the overall death rates were not initially reduced very much. Clinical trials in which the influence of treatment on time to death is of prime interest should rarely be undertaken unless *either* there is some hope that the death rate can be halved, *or* the trial will be able to continue until at least 100 patients have died, which will usually require the admission of well over 100 patients. There are exceptions, of course (the chief one being when the disease is so rare that large trials are impossible, even if many hospitals collaborate), but it is unusual for the comparison of the times to death of a smaller number of patients to be of much value, unless (as might be the case if no large series of such patients had yet been reported) the chief point of the trial is not the comparison of 2 treatments but rather the study of the natural history of the disease. Moreover, the patients may need to be entered over a reasonably short time, if the trial is not to be overtaken by results from other trials. Because of this, in all but the commonest diseases, no one physician or even hospital department will be able to complete alone a successful trial studying survival duration. Cooperation between independent physicians in different hospitals is often essential.*

It would undoubtedly be better if the organizers of most of the hundreds of different small trials currently in preparation or progress at single centres around the world attempted to secure the collaboration of colleagues at other centres in randomizing patients into their trials. Sometimes quite a small organizational effort can double or more than double the size of a given trial by recruitment of other centres at which, otherwise, no sort of a trial would have been running. It is wrong for research-oriented centres to disdain such collaboration from centres which are not research-oriented, even if the standards of medical management are presumed not to be quite as good as at the research-oriented centre. In fact, since the success of a trial depends so strongly on the numbers of patients randomized, it should perhaps be emphasized when soliciting collaboration that all physicians who do collaborate wholeheartedly will be full co-authors of any eventual publications.

4.—*What treatment schedules should be compared?*

**A positive result is more likely, and a null result is more informative, if the main comparison is of only 2 treatments, these being as different as possible.**

The chief point is that the question answered must be the most important question the investigators can think of, which they could answer by a clinical trial. A lesser study of an important question is usually of more value than an excellent study of a trivial question. Next, discover who else is answering the same question; the EORTC and UICC keep current lists of the hundreds of cancer trials currently in progress. The American NCI keeps lists of all current trials of immunotherapy (Windhorst, 1976), and has commissioned a compendium which is supposed to be updated every 3 months of all clinical trial protocols, including those on the European lists; the first issue of this has just appeared (Smithsonian Corporation, 1976). Become familiar with these lists at the earliest possible stage of your planning, but remember that many listed trials are so small or poorly controlled that the questions they ask may still

* In such cooperative studies, there is no assumption that the type of patient or quality of management at one centre is comparable with that at another centre. It is only assumed that if a treatment is of medical value, then this will be true at every centre, and although the treatment difference actually observed at an individual centre may be randomly obscured or even reversed, the overall sum of all the separate treatment differences, one from each centre, should point in the right direction and the separate differences should thus reinforce each other, even if most, or perhaps all, of the separate differences are not individually significant.

need to be answered by a large, randomized trial.

Only a very limited time may be available before results of other trials intrude, and lead to strong pressures to modify treatment details or even completely to abandon certain schedules. It is thus usually more efficient to compare only 2 treatment schedules in any trial, since this gives the maximum chance of being able to draw some definite conclusions before the schedules have to be modified. (In this context, of course, 1 of the 2 treatment schedules might be " no treatment ".)

Moreover, these 2 treatments must be sufficiently different from each other for it to be medically plausible that the death rate (or the rate of whatever type of event is of chief interest) on one *could well* be very substantially lower than on the other. If it is not plausible that a slight difference in radiotherapy fractionation or in drug dosimetry could have a substantial effect on the outcome, then clinical trials of such slight differences are unlikely to be useful. We are not arguing that the answer has to be known before the trial could be designed, of course—merely that treatments that *could well* differ substantially should be compared.

Many clinical trials yield null results (*i.e.* they find no statistically significant differences between the groups of patients given different treatments) and it is a mark of good trial design that a null result, if it occurs, will be of interest. If you are trying out a new drug, give the biggest dose of it that you safely can, so that nobody can say, if you get a negative result, that if only you had given more it would have worked. (A drug trial is always a trial of the drug in the particular dose and manner given, not a trial of the drug *per se*.) If you are checking other people's work, repeat their protocols exactly, so that they cannot suggest that minor irrelevant differences between the conduct of your trial and theirs were actually relevant. If you are testing whether a reduction in the amount of therapy is possible, reduce the therapy as much as you dare, and so on. Negatives in such trials are of much wider interest than negatives in less extreme comparisons. When designing a trial, one usually has the possibility of discovering a clear difference foremost in one's mind, but it is a useful exercise to force oneself to answer the questions " What medical value will these results have if both treatment groups fare equally well? Could the trial design be altered to make a negative result, if it occurs, even more valuable? "

An agent which is therapeutically effective when given in one particular way may be much less effective when given at a different dose level or with different intervals between doses. This makes it difficult to use results from a clinical trial to discover which of 2 different agents is absolutely best, for one of the agents might have been better if used differently. (This difficulty can be avoided if each agent is sufficiently well understood for the investigator to be confident that it is being used optimally, or almost optimally, but this is unusual.) In the particular case of a trial intended to discover the better of 2 cytotoxic anticancer agents, both of which damage normal, as well as neoplastic, cells, the agent to be preferred is usually the one which *for a given degree of damage to normal cells* produces the greater anticancer effect. This agent can usually only be identified by a clinical trial if the schedule for each agent produces a similar degree of damage to normal tissue. When testing actinomycin *versus* vincristine, therefore, perhaps the total doses to be given should not be specified in milligrams, but rather in terms of the approximate degree of myelosuppression to be attained. Likewise, when comparing 2 forms of radiotherapy, perhaps each should be given until a certain degree of damage is observed in adjacent normal tissue. When designing (or interpreting) a trial which compares 2 different anticancer agents, this question should be carefully considered.

### 4A.—Secondary studies

So far, we have argued that a question is more likely to be successfully answered by a clinical trial if it can be answered by comparing just 2 alternative treatments and no more, those treatments being as markedly different as possible. However, if 2 largely unrelated questions can be posed (*e.g.* " Do platelet transfusions help remission induction? " and " Does regular maintenance cytotoxic therapy during remission help? ") then both can be answered by a single clinical trial. At presentation, the patients are randomized as to whether or not they get platelets, and those who achieve remission are then randomized as to whether or not they get maintenance therapy. Actually, more than 2 questions can be answered at once: for example, we could have, in addition, randomized the patients who achieved remission, so that half received immunotherapy and half did not (so one-quarter would have received immunotherapy *and* maintenance, one-quarter would have received immunotherapy and *no* maintenance, *etc.*).

If a clinical trial is large enough to be scientifically worthwhile, the efforts of recruiting the large number of patients entered into it, and of following them up, are substantial. The extra scientific pay-off from answering extra questions is usually well worth the slight extra organizational effort involved in multiple randomization, as long as you don't try to answer 2 very strongly related questions in the same trial. Once a substantial clinical trial is definitely to be undertaken to answer one particular important question, explicit attention should next be directed towards identifying other, probably lesser, questions that the trial could easily answer concurrently by multiple randomization, as long as the trial is not made so complex that potential collaborators are deterred from participation in this (or future) trials.

Trials may also be used as a source of information about the natural history of a disease, quite independently of the therapeutic question which is being asked, perhaps by measuring or recording something at presentation and studying its correlation with some other recorded factor, or with time to death or to whatever endpoint is of interest, or perhaps by making serial records of something so that its pattern of change over several months can be studied. It may be wise to make such studies optional, in that some centres will do them for all patients while other centres will not do them at all, otherwise the number of patients randomized may be reduced.

Finally, the commonest reason for deviations from schedule in cancer trials is probably treatment toxicity, necessitating that less than the specified dose be given or that courses of treatment be delayed. Specification of schedules should therefore include details of what to do if undue toxicity emerges: practicable, flexible schedules avoid many " deviations " and are more relevant to real medical practice, as long as the reasons for " flexibility " are clearly formulated.

5.—*Significance levels* (" *P-values* ")

### " *P*=0·05 " does not mean " the probability that the treatments are equivalent is 0·05 ".

There are fewer sources of error and bias in a randomized study than in a non-randomized study, but even with proper randomization misleading results can emerge.

If a group of patients treated one way does better than another group which was treated in another way, there are 2 possible explanations for this: either the first group got better treatment, or the first group contained disproportionately many patients who would have done well anyway, even if they had been treated in exactly the same way as the second group. Unfortunately, division of patients into 2 treatment groups by randomization is no guarantee that the 2 groups have equal proportions of patients with good and bad prognoses, and so, even in a randomized trial, spurious differences between treatments will sometimes arise. The whole elaborate clinical trial machinery of randomization, objective assessment, avoidance of losses to follow-up, and so on, however, ensures that *if* a substantial difference emerges between the average outcomes in the 2 treatment groups, *then* we can calculate the probability of getting a difference at least as substantial as this by chance alone if the 2 treatments are in fact equivalent. This probability is called the "significance level", or "$P$-value". Even if the 2 treatments are exactly equivalent, our random allocation may, by chance alone, put more of the good-prognosis patients on to one treatment than on to the other. The exact meaning of the familiar abbreviation "$P < 0{\cdot}05$" is thus "the patients in one treatment group have fared better than the patients in the other. *If* there is no difference between the medical effects of the 2 treatments and the only cause of differences between the treatment groups is the chance allocation of more good-prognosis patients to one group than to the other, *then* the chance of one treatment group faring at least this much better than the other group would be less than 0·05, *i.e.* less than a 1 in 20 chance."

It is worth the effort of understanding this convoluted statement, since the logic of it contributes to so much modern medical research. (It does not, for example, mean that the probability that there is no difference between the treatments is 0·05.) A significance level is, it may be seen, an extremely indirect answer to a physician who simply wants to know which treatment works best (especially since events with probability less than 0·05 are really quite plausible: throwing double 6 with a pair of dice, for example), but it is better than no such answer at all. To calculate the significance level, it is not necessary that exactly similar proportions of patients with bad, medium and good prognoses were allocated to each treatment. What is necessary is merely that the *probability* of a trial patient getting one or other treatment be independent of whether that patient has a bad, medium or good prognosis, and that the standard of assessment of success or failure be independent of treatment. This means that stratified allocation (which is discussed later) is not necessary, but randomized allocation is.

6.—*Further reasons for large trials*

**A given $P$-value in a large trial is usually stronger evidence that the treatments really differ than the same $P$-value in a small trial of the same treatments would be.**

There are hundreds of well-conducted clinical trials now in progress, which are comparing 2 essentially equally effective treatments. Unfortunately, at least 1 in 20 of these null trials will report a misleadingly significant ($P < 0{\cdot}05$) difference. Conversely, many of the trials which are now comparing 2 genuinely different treatments will not observe a "significant" difference. How, then, should claims of "statistical significance" be assessed, when it is common experience that many early claims are later refuted? In classical statistical theory, only 2 criteria—the $P$-value and the intrinsic plausibility of the claim which the $P$-value supports—are supposed to be balanced, but in assessing these to decide whether or not the 2 treatments really differ at all, the size of the trial is an additional, independent, third criterion. Let us consider this in the context of a trial where a new treatment is being compared with, as control, a standard treatment.

Nowadays, for every trial that compares 2 treatments which are substantially different, there are probably 5 to 10 "null" trials in progress comparing 2 treatments which are almost equally effective. Moreover, even the "substantial" differences are by no means so substantial that small trials can reliably detect them: it might be, for example, that when 50% of the control patients are dead, only 33% of the patients receiving

TABLE I.—*Numbers of Trials Currently in Progress that will be Statistically Significant*

| Planned trial size (unless an early treatment difference stops the trial when smaller than this) | Expected proportion dead in one treatment group when half the patients in the other group are dead | Postulated no. of such trials now in progress world-wide | Given these postulated numbers in progress, approximately how many of them will be: (a) Non-significant | (b) Significant at $P < 0{\cdot}05$ or better |
|---|---|---|---|---|
| About 250 deaths (*i.e.* an entry of some hundreds of patients) | 50% (no real difference) | 100 | 95 (right) | at least 5* (misleading) |
| | 33% (treatments really differ) | 20 | 1 (misleading) | 19 (right) |
| About 25 deaths (*i.e.* an entry of some dozens of patients) | 50% (no real difference) | 1000 | 950 (right) | at least 50* (misleading) |
| | 33% (treatments really differ) | 200 | 150 (misleading) | 50 (right) |

* Even the most rigorously designed, executed and analysed studies have a 1 in 20 chance of a $P < 0{\cdot}05$ false positive. The less rigorous the study, the greater the chance of a false positive, and in practice perhaps 10% or more of all studies yield such false positives. A major error, premature termination, is discussed in Statistical Note 1 on page 610.

the new treatment would be expected to have died. A difference of this magnitude has over 95% chance of being detected in a trial in which hundreds of patients are randomized and about 250 of them die, but only a 25% chance of being detected in a small trial in which dozens of patients are randomized and about 25 of them die.

We need now only to postulate a few reasonable numbers to see the effects of this situation (Table I). Three immediate consequences of the numbers in Table I may be discerned:

(i) A large proportion (pehaps even the majority) of reports of statistically significant treatment differences in small trials are misleading, and no real differences exist. (This is not true of trials which are planned to be large*.)

(ii) If a small trial compares a new treatment which is so effective that it prevents one-third of the deaths with a control treatment then it will probably fail to reach statistical significance. (This is not true of a large trial.)

(iii) A serious bias arises because most of the interesting therapeutic questions are being studied simultaneously by many trials: for example, 400 are in progress, studying the immunotherapy of malignant disease (Windhorst, 1976). Any trials, large or small, in which patients given the new therapy fare significantly better, will be published and publicized, but few trials which discover no difference will achieve wide attention, especially if they are small. The UICC are attempting to rectify this situation by publishing each year, in their Technical Report series, a list of all trials currently open or recently closed, specifying reasons for closure. Despite this effort, the literature remains biased. It is an inevitable bias, but one which can be circumvented to some extent by restricting attention to trials so large that they would be published whether or not a difference was observed. A further consequence

* However, trials comparing genuinely different treatments which are planned to be large may yield such striking results when still small that they are stopped and published. This would increase the number of genuine differences in published small trials at the expense of the number of genuine differences in published large trials. The effect is substantial only if the real treatment difference is sufficiently extreme for there to be a good chance of its detection in a small trial, which will often not be the case.

of publishing only the trials where large differences are apparent is that, when it is claimed that a new treatment is better, even if this claim does eventually turn out to be true, the magnitude of the benefit associated with the new treatment will almost always be over-estimated by the first few published studies.

In summary, collaboration between centres, which are now independently organizing small trials to answer similar questions, would be greatly in the general interest, as long as the large trials thus initiated are competently organized. Once a moderately large trial has been organized, however, how extreme must the $P$-value be before the results can be believed?

7.—*Prior opinions*

**It is proper to combine prior opinion and knowledge with $P$-values to guess the truth.**

Consider a publication describing a moderately large randomized clinical trial, organised by reputable investigators, which purports to show that a certain treatment is useful. Suppose that, before you saw these results, you already thought that this treatment probably worked. Even if the reported difference is not statistically significant, you would become more definite in your opinion, while if a $P$-value of 0·05 is reported, there would be almost no doubt left in your mind that this treatment works.

Suppose, instead, that before you saw these results you had no opinion, but on reflection the claim seems reasonable (*e.g.* systemic chemotherapy in Stage II breast cancer). Now, a $P = 0{\cdot}05$ result would not in itself be convincing, although it would make you more receptive to future such claims; a $P = 0{\cdot}01$ result would be difficult to dismiss; while a $P < 0{\cdot}001$ result would be extremely convincing.

Suppose, finally, that, had your opinion been sought before reading the published report, you would have thought that there was little prospect of such a treatment being of value. Now, a $P = 0{\cdot}05$ result would leave you almost as sceptical as before; and although a $P < 0{\cdot}001$ result would change your mind, you would still retain a secret little doubt.

Different investigators will not, of course, agree as to what is plausible, and so in published reports more emphasis is often placed on $P$-values ($P$-values should be subject to public agreement) than on opinions. However, different readers will judge a claim differently, and the heterogeneity of their prejudices is a useful safeguard against collective error.

Conversely, there are many instances when people should have been guided by positive or by null findings in randomized trials, but were not; the cardiotoxicity of certain oral hypoglycaemic agents has been strongly suggested by a large randomized trial, but this finding is still dismissed by many diabetologists. They may be right to dismiss it, but, if they are not, many avoidable deaths are being caused.

8.—*Non-significant differences*

**What may be inferred if there is no statistically significant difference between two treatments?**

An exact answer requires statistical expertise, but adequate approximations are easy to construct. Two things are important: the size of the trial, and what was observed in it (approximate equality or an appreciable difference which nevertheless was not significant).

In a small trial, a non-significant result yields almost no information, except that if there was an appreciable difference then the apparently better treatment is unlikely to be much *worse* than the other treatment, although it could well be a little worse. (This may be a sufficient answer, if the apparently worse treatment is very toxic or expensive.) If approximate equality was observed in a small trial, there could still easily be a 2-fold disparity between the death rates on the 2 treatments, while if one did appreciably better than the other, then maybe there is no real difference, or maybe the apparently better treatment is 2, 3 or 4 times as good. Such unreliable information is of much less value than data from a larger study would have been.

In a large trial, involving about 250 deaths, if approximate equality was observed, then there could still be a 20% treatment difference in either direction, while if one group fared appreciably better, then the treatment they received might really be no better, or it might really prevent 10, 20 or 30% of deaths. Thus, even null results from large trials have considerable uncertainty attached to them, although they are of more value.

Such null results are so vague, and significant results indicate the magnitude of any real difference that may exist so imprecisely, that it should cause no surprise, when several studies of the same question are compared, to discover that some are highly significant while others are nowhere near statistical significance. We have already seen that " significantly different " is not synonymous with " different "; still less is " non-significant " synonymous with " identical ". In fact, no statistical argument can ever demonstrate 2 treatments to be identical; all one can do is to say what range of differences is consistent with the observed data.

### 9.—*Treatment allocation ratio*—1:1 *or* 2:1?

**Unequal allocation may be best.**

In any clinical trial report in which a new treatment is claimed to be effective, readers will assess the claim, not only in the light of the comparison with the trial control group, but also by comparison with their own experience and ideas as to what is plausible. This is, of course, right and proper; but for comparison with other data, the larger the group who received the new treatment the better. It may, therefore, be advisable, when comparing " new " with " old ", to randomize not in the ratio 50:50, but perhaps in the ratio 60:40 or 67:33 (2:1). Randomization in the ratio 2:1 may also be preferred, if one treatment is much more expensive or inconvenient than the other. The chance of obtaining a statistically significant difference between the 2 treatments is not reduced much, as long as the chosen ratio is less extreme than 70:30*, and the increase in the size of the " new " group that can be reported may easily outweigh this power loss.

Suppose 2 minor variants of a new treatment are possible, and we adopt equal 3-way randomization between control and the 2 variants. Comparison of the control patients with all patients given the new treatment, irrespective of which variant they received, gives us an efficient 2:1 trial of whether the new treatment is any better than control. If it is, then as a slight bonus we already have a small randomized study comparing 2 variants of it.

Alternatively, if when arranging a collaborative trial between 2 treatments there is a definite difference of opinion about a lesser detail of one of them, collaboration may be saved by the proposal of 3-way equal randomization at all centres, which will still yield a useful 2:1 study of the main question.

### 10.—*Randomized controls or " historical controls "?*

**Assessing a new treatment solely by comparison with past experience can be misleading; at least one-third of current patients should be randomized controls.**

If a clinical trial is simply designed to

* Compared to a 50 : 50 randomization, 60 : 40, 65 : 35 or 75 : 25 randomizations entail reductions in the chance of obtaining a statistically significant difference which are approximately equivalent to the reductions produced by eliminating 4%, 9% or 25% of the patients from the trial.

discover whether a new treatment is better than the standard treatment, and to answer no other questions, the normal practice is to randomise (1:1 or perhaps 2:1) and to compare the fates of the 2 groups. However, some people prefer to put all the patients on to the new treatment and merely to see if they fare better than previous " such " patients. The advantages are that randomization, which is sometimes difficult to explain to the patient, is avoided; the group getting the new treatment is bigger than it would have been, since it now includes all the patients who would have been controls; and that the " control " group is bigger still, comprising many other patients from previous series. The overwhelming disadvantage is that there may very well be systematic differences between the old series and the treated series due to changing referral patterns to the study centre, changes in supportive therapy, changes in the skill of the doctors, or subconscious (or even conscious!) selective biases: for example, the omission of a few old or moribund patients from the new series can make a big difference to the overall outcome.

Byar *et al.* (1976), in a readable paper on this and other aspects of clinical trial methodology, give a nice example of 2 large series of prostatic cancer patients, selected by the same criteria at the same centres, both given placebo yet differing substantially in survival. Likewise, S. J. Pocock has collected 19 unselected other instances where consecutive trials in the same malignant disease at the same collaborating centres carried over a common treatment arm from one trial to the next. The results when one arm was compared with the supposedly identical arm in a later trial were often materially different; 10 of the 19 2-tailed $P$-values were less than 0·2 (as opposed to 4 expected by chance), and 3 were less than 0·02.

From the point of view of the reader of a publication describing a clinical trial in which a recent series is only compared with previous experience, there should always be grave suspicion that maybe initially the 2 groups of patients were systematically very different, or that the treatments they were given also differed in ways other than the treatment difference which is claimed to be the only important variable. (Changes in the standards of supportive care, or in the ability to use a potent drug successfully, can easily occur.) A prudent reader would, perhaps, say that the chance of a substantial systematic difference due to conscious, subconscious or accidental bias is at least 5 or 10%, and so no $P$-value less than 0·05 can in principle be obtained from such a study, if by " $P$-value " we mean the probability of such results emerging if there is no real difference between the treatments.

Such studies can be of some value, of course. If the new treatment cures almost everyone who would previously have died, historical controls may suffice to demonstrate this adequately. Also, of the $P \simeq 0{\cdot}05$ differences reported from the hundreds of randomized trials currently in progress, a good proportion are artefacts of chance, and comparison with other series can help sort out which are real. If the randomized controls have fared a lot worse than expected on the basis of previous series, this is probably one of the bogus significant results, while if the randomized controls seem to have fared ordinarily, the claim is more plausible. This use of historical series to cast doubt on current claims is most valuable, and should be more widespread. Finally, a physician convinced of the merit of a new treatment cannot ethically randomize his patients, and must use historical controls: a proper function of his research might be to convince any sceptical colleagues that a large randomized trial should be undertaken.

However, unless a trial is seen by the investigators only as a " pilot " trial, a precursor to a future randomized study, it is probably wise to randomize, and Chalmers (1975), in a delightful 3-page paper, argues that even pilot trials should be controlled by randomization. A reasonable compromise for someone intent on a major study using only " historical controls " would be (unless the new treatment is so confidently preferred that randomization is felt to be unethical) to randomize in the ratio 2:1. Two-thirds of the patients are still available for comparison with whatever " historical controls " are preferred, while people who only believe results from randomized trials have almost as substantial a randomized study to examine as if the more ordinary 1:1 randomization had been adopted.

Inevitably, however, many situations will arise where only historical comparisons are available to a reader, and no large randomized study will be available in the near future. Since a common bias in historical comparisons is the inclusion in one series of more of the acutely ill patients than in the comparison series it may be of value to ask " Among the survivors 6 months after entry in both series, was there subsequently any appreciable difference in survival?" and, if not, to suspect strongly that the 2 series of patients differ not in efficacy of treatment but rather in efficacy of exclusion of the acutely ill from one series. Comparisons with a delayed start such as this are irrelevant if the new treatment is chiefly concerned with an early acute phase of the disease, but may be of value in casting doubt on claims for treatments which, if effective, would be expected to be of continuing therapeutic activity.

" Blind allocation ", in which the patient is unaware of which treatment he is receiving, and " double-blind allocation ", in which both the patient and physician are unaware, are sometimes excellent devices for helping ensure that response is assessed objectively, and whenever assessment of the response of interest is subject to much uncertainty it is worth considering whether the treatments could be formulated effectively in ways which appear indistinguishable to the patient, the physician, or both. Alternatively, " blind assessment " of a subjective response by another physician, who really is unaware of the treatment being given, may be of value. A subjective response, such as " disease stasis " or " escape from disease stasis ", in solid tumour therapy should either be avoided, or assessed in as objective a manner as possible.

11.—*Treatment allocation*

**Balanced randomization at the latest possible time is recommended, with no stratification; Appendix 2 gives practical details of how this is done.**

At the start of a trial, each participating centre may be given an ordered set of sealed envelopes. A preferable technique is to keep the envelopes at a central office and to make the separate centres telephone for instructions when a new patient is to be randomized. This uses more administrative time, but it does avoid all suspicion that any form of cheating has occurred, and it leaves no doubt at the central office about exactly who has and who has not been admitted to the trial. Each envelope contains the instruction as to which treatment schedule to follow for a particular patient. When a patient who appears to satisfy the admission criteria is found, he is formally admitted, and *only then* is the envelope opened to discover his treatment.

If randomization is to be by reference to a central office, a simple randomization list may be constructed instead of a set of envelopes, as long as this list, which specifies the order in which treatments will be allocated throughout the trial, will never be seen by any physicians responsible for patient entry, including any trial organizers who also treat patients.

If the treatment to be given to the next trial patient is known before the decision whether to admit him is made, this decision may well be influenced by the knowledge of what treatment he was to receive. If this happens, the groups of patients on the different protocols may differ systematically, and consequently the trial result may be of little value. It is not permissible to enter patients and then withdraw them from the trial if the treatment allocated is felt to be inappropriate: once admitted, patients must be followed up and included in the final report of the trial. If *any* of the treatments a patient might get are felt to be inappropriate, give that patient whatever treatment you think best, but *don't* include him in the trial. In other words, before randomizing a patient into a trial, check that you would be prepared to give him any of the treatments, that he would be prepared to receive any of them, and that there are no geographical or social reasons which make any one of the possible treatments impracticable.

In a randomized trial comparing 2 treatment schedules for patients whose disease has first been controlled by standard therapy, there may be an initial period during which the treatment a patient should receive is the same no matter what schedule will eventually be specified for that patient. For example, in the MRC trial of immunotherapy for myeloid leukaemia, the patients are first treated by chemotherapy in order to induce partial control of the disease: many patients do not achieve such remission of disease, but those who do are then randomized between continued chemotherapy and continued chemotherapy plus immunotherapy. Randomization could occur when the patient is first diagnosed, or at any time during the early intensive phase which is common to all patients, or not until remission has been completely achieved. The advantage of early randomization is that it is easier for doctor/patient relationships if the doctor knows as soon as possible how he will treat his patient. The grave disadvantage of early randomization is that unbiased analysis of most such trials can usually only be guaranteed if all deaths occurring in either treatment group after randomization count against that group—even including deaths during the early period, when patients in both groups should be being treated identically! If, therefore, randomization takes place well before the patients in the 2 groups have to be treated differently, chance differences in what happens in the early (common) period may dilute real differences in the later period. Randomization should, therefore, usually take place as late as possible, and analysis should preferably compare the totality of post-randomization mortality in one group with that in the other. These rules also apply in clinical trials of whether to stop regular anti-cancer treatment in patients who have been apparently free of disease for some time. It is preferable to count the total number of post-randomization deaths in each group, even if randomization occurred after slightly different disease-free periods in different patients. It is therefore generally preferable to randomize as late as possible, so that almost immediately after randomization the patients in different groups will start to receive different treatments. Likewise, in trials comparing different treatments for the relapse of previously controlled disease, wait until relapse actually occurs before randomizing.

The order in which the treatment schedules appear in the sealed envelopes should ideally be:

(1) Unpredictable, in the sense that the physician may know that the overall ratio is 2:1, 1:1, 1:2 or whatever, but given this, he has no further idea what the next envelope contains.
(2) Balanced, so that when the trial stops, the numbers of patients on the 2 treatments are roughly in the desired ratio.
(3) In addition, it would be an advantage if the patients with good and bad prognoses were also each balanced in the same ratio between the treatment groups.

A variety of allocation schemes can be devised, ranging from simple coin tossing to elaborate " stratification " schemes in which a separate series of envelopes is provided for each of several prognostic categories of patient*. However, proper statistical methods, such as those which will be introduced later, make due allowance when comparing 2 treatments for what was initially known about each patient. (This may be thought of as " retrospective stratification ".) If proper methods of analysis will be used, stratified entry makes little difference, and it is usually completely satisfactory to have a single series of envelopes for all patients, and not to bother to " stratify " in any way. This is discussed further in the next section. How *should* the sequence of treatments in these envelopes then be determined? The difficulty is that, unless the numbers are large, the requirements for (1) unpredictability and (2) balance are to some extent incompatible. Because of this, some statisticians say " Randomize: that's the only way to achieve complete unpredictability " (which is true), while others (like us) say: " Constrain the randomization to keep the proportions allocated to the 2 groups reasonably close to your chosen proportions." (Statistical Note 2, on p. 611, discusses, but does not recommend, other, more complex, allocation rules.) The disadvantage of randomizing if your chosen proportions are, say, 1:2 is that you may end up analysing a very lopsided trial with 5 out of 30 on one treatment instead of an intended 10 out of 30. For this reason, complete randomization should not usually be used, unless the intended allocation ratio is 1:1, in which case it is fairly safe, even though pseudo-randomization may still be preferred: see Appendix 2. The disadvantage of pseudo-randomization is that the physicians may see through the scheme, work out the next treatment, and so admit different types of patient to the 2 treatments, but we have never known of this happening.

The practical details of both methods are trivial: to produce 100 envelopes randomized in the ratio 1:1, you toss a coin 100 times, getting a series of letters A (for heads) or B (for tails), and then you make up the envelopes in the order specified by your list, keeping the list to check that the physicians use the envelopes correctly and obey them. To produce 100 envelopes pseudo-randomized in the ratio 2:1, 1:1 or 1:2, you may use the methods described in Appendix 2. In a multi-centre trial, it is slightly preferable to have a balanced allocation list (see Appendix 2) for each separate centre, especially if the number entered by some centres is very small. This form of stratification involves no extra trouble whatever for the participating centres, and actually helps maintain their interest, by ensuring that each centre is called on to administer each treatment.

12.—*Should " stratified " allocation be envisaged?*

**If, during analysis, initial prognosis will be allowed for while the different treatments are being compared, there is hardly ever need for stratification at entry in large trials.**

As long as good statistical methods, such as those given in Part II of this paper, are used to analyse data from clinical trials, there is no need for randomization to be stratified by prognostic features. Moreover, if the organizational complexity of it deters any collaborators from entering patients during busy clinics (or deters

* If each of these separate series of envelopes is completely random, no improvement in balance whatever is obtained by such " separate randomization ": the treatment prescriptions in each particular such series of envelopes must therefore alternate, or have some other such constraint imposed on them, if stratification at entry is to have any effect whatever.

them completely from collaboration in this or in some future trial), positive harm will have been done by the initial stratification. These views are not generally accepted, so in this section we argue them at greater length than many readers will wish to follow. If you agree that stratified randomization is usually unnecessary (except, perhaps, in very small trials), or if you are indifferent on the matter, skip this section.

Usually, for purposes of statistical analysis, the patients will be subdivided into a few strata, defined retrospectively from those features (*e.g.* age or stage) which are eventually found to be really relevant to prognosis. The points of subdivision of each such feature can be chosen in the light of the actual data, to make the prognostic discrimination most sharp, and certain groups with a similar prognosis may finally be merged. The patients within one stratum will then be compared with each other, to see if treatment appears to have been beneficial, and the comparisons within each stratum will finally be combined to give a single overall $P$-value for the effects of treatment adjusted for initial prognosis (see Appendix 3 in Part II for a worked example).

If the distribution of treatments is similar in each stratum, no actual bias is corrected by such " retrospective stratification ", but the fact of comparing like with like makes such an analysis slightly more sensitive. Suppose now, that we stratify at entry, randomize with " balanced " random numbers within each initial stratum (or assign treatments by alternation within each stratum), and suppose further that, by luck and good judgement, the initial strata are all subsets of the retrospective strata which we devise during the eventual statistical analysis of the data. *Retrospective stratification will still have to be undertaken, for if no allowance is made in a statistical analysis for the fact of stratified entry, the calculated P-values are not extreme enough.* The only advantage gained by stratification at entry is that, within each retrospective stratum, reasonable balance between the numbers on each treatment will automatically be achieved, and a wasteful situation, where almost all the patients in one retrospective stratum happen to get the same treatment, is avoided. This advantage, however, is largely illusory, unless the trial is very small. The improvement in the sensitivity of a clinical trial to be expected from achieving perfect balance between the numbers on each treatment in each restrospective stratum, instead of letting them be defined by chance, is just that to be expected from randomizing a single extra patient into each retrospective stratum. (This is proved in Statistical Note 3 on p. 611.) However many initial strata are defined, only a few retrospective strata will be needed, and so the expected benefits from initial stratification are slighter than would intuitively be expected; indeed, if the organizational complexity of stratification at the time of randomization reduced collaboration at all, a net loss of efficiency would be the likely result. This objection does not, of course, apply to stratification by centre in a multi-centre trial.

### 13.—*Exclusions, withdrawals, losses, and deviations from treatment*

**Rigorous entry criteria are not necessary for a randomized trial, but rigorous follow-up is. Even patients who do not get the proper treatment must not be withdrawn from the analysis.**

Individual physicians will probably have, for certain of their patients, a definite preference for one or other (or none) of the trial treatments. When this happens, the patient cannot ethically be admitted to the trial in case he gets the " wrong " treatment: he must be excluded from the trial, and be given the treatment thought best for him, even if there is little objective basis for this preference.

It is worth including in the trial protocol specific instructions against randomizing patients who are unlikely to tolerate, or who may be unable to receive, *any* of the treatment schedules (*e.g.* through having " bad " veins, and thus not able to receive prolonged i.v. therapy), who are extremely old or extremely young for the disease, who seem unlikely to cooperate, who live so far away that regular treatment will prove difficult, or whose disease seems likely to take an abnormal course. Certain of these patients may teach one a lot about the disease, but not through inclusion in a clinical trial. Also, if the disease process is very long, it is best to restrict admission to patients who are likely, as far as one can tell, to continue to attend the same hospital throughout the course of their disease.

It is also best to restrict collaboration to centres thought likely to continue to collaborate seriously for a few years, for if, in a multi-centre trial, some centres lose interest during the first year and start giving trial patients all sorts of different treatments, or stop supplying the necessary follow-up information, the result can be progressive collapse of the whole study. This may be preventable if deviant centres are quickly expelled or quickly asked to reform, but this requires up-to-date monitoring of the study by a group with some moral authority.

In a clinical trial, there are 3 distinct categories of missing patients: those who are excluded before randomization; those who, despite having been randomized, are deliberately withdrawn from the trial as though they had never been entered; and those who are inadvertently lost during follow-up, and whose experience can be included in the statistical analysis only up to the date of loss. There is no agreed terminology for these 3 categories, and we will refer to them as exclusions, withdrawals, and losses, respectively (see Fig. 2).

### 13A.—Exclusions.

Exclusions, whether for serious reason or a whim, do not bias the randomized treatment comparison (in fact, they do not enter into it at all), and they are therefore acceptable under all circumstances. It is, however, preferable to define roughly the

FIG. 2.—Exclusions, withdrawals, and losses.

reasons used for exclusion and inclusion, so that other workers can judge your average results and compare them with other series.

The participating centres may agree to make a list of every patient presenting at that centre with the disease being studied, noting whether that patient was randomized and, if not, why not. Such lists may help characterize the patients being studied in the trial, which may be useful but is not logically necessary in a randomized study. Such lists may also, perhaps more importantly, help prevent the rate of entry falling off as enthusiasm wanes or staff changes occur at the various centres.

### 13B.—Withdrawals.

Sporadic losses and, particularly, withdrawals *can* bias the results, and adopting an explicit policy towards withdrawals and losses should be part of the design stage of a trial. One excellent policy is to accept no withdrawals under any circumstances. This may not be satisfactory, however, if the differential diagnosis of the disease is difficult. For such diseases, it may be best for the information collected from each patient *at the time of randomization* to be reviewed centrally, in ignorance of the treatment schedule to which the patient was allocated, so that patients thought at this central review not to satisfy the agreed trial criteria can be withdrawn as if they had never been entered, without in any way biasing the treatment comparison. If the initial diagnostic tests are known always to yield, when results eventually emerge from them, simple *unequivocal* yes/no results, then it would be acceptable to include in the trial design the instruction that the diagnostic test should be done at randomization for every patient (or at least for every patient for whom the differential diagnosis is uncertain), and that when the results become available those patients tested at randomization and found to have the wrong disease should be withdrawn by the responsible physician, with no reference to the trial secretariat.

If there is to be a central blind review, it may actually take place long after randomization; indeed, there is some advantage in reviewing a large number of the patients simultaneously to ensure uniformity of judgement. If the review occurs after the trial has closed, all patients can be reviewed together.

In the foregoing rules (which should be adopted whenever possible), observations made at the time of randomization are used to determine without bias who will be withdrawn. Unfortunately, for example, acute undifferentiated lymphoblastic leukaemia might be mistaken for myeloid leukaemia by all criteria that can be recorded at presentation, and the misdiagnosis might not be recognisable until partial control of the disease has been achieved. The previous rules will not then apply, and there are then only 3 options for the design of such trials:

(1) Do not randomize patients unless or until the differential diagnosis is unequivocal. (This may lose some appropriate patients from the study.)
(2) Randomize some or all of those in whom the diagnosis is doubtful, withdrawing any who subsequently proved to have the wrong disease as if they had never been randomized.
(3) Again, randomize some or all of those in whom the diagnosis is doubtful, but leave all randomized patients in the final analysis, even those whose diagnosis was revised and whose treatment consequently altered. (This is always valid, and is sometimes the only valid policy, but it is somewhat artificial.)

Option (2) is medically preferable, as long as it is statistically valid, and it is more widely valid than most statisticians believe. It is true that, if one treatment keeps patients alive longer than the other treatment does, then there may be more opportunity for diagnostic revision and hence withdrawal, but this is unlikely to completely obscure the merits of the better treatment. What determines whether (2) is acceptable therefore depends chiefly on what might be expected if the two treatments have equivalent effects on survival duration. In this case, (2) is only invalid if the different treatments will affect the probability of diagnostic revision differently, and this will be the exception rather than the rule.

Whatever rules are adopted to deal with the problem of possible misdiagnosis, they should be written into the design of the trial and not invented *ad hoc* during the statistical analysis to exclude an unwanted patient or two from a particular group.

### 13C.—Losses and deviations.

Patients who move away from the centres where they were admitted to the trial should not be allowed to disappear from the trial. If possible, their fate should be discovered, perhaps by extensive telephone enquiries, letters, or even special visits by research assistants. (The MRC leukaemia trial policy is to accept no reason, other than permanent emigration from Britain, for loss, and so they try to avoid including foriegn nationals in their trials.)

It is sometimes suggested that if a substantial deviation from the allotted treatment occurs, that patient should not be included when the final comparison of treatments occurs (or should be included only up to the date of deviation). This is seriously wrong, as the group which deviates from one protocol and the group which deviates from the other protocol may be so different in their chances of long survival that the treatment comparison in the remaining patients will be severely biased. Disagreement about this point is perhaps the chief source of misunderstanding between statisticians and clinicians about the logic of trial design. To clinicians who disagree, it might be pointed out that including all the deviants can only affect the conclusions appreciably if the deviants are more numerous in one treatment group than in another, and grossly different in survival duration from the protocol adherents, but in this case exclusion of them is not valid. Withdrawing protocol deviants from the statistical analysis is therefore either *irrelevant* or *invalid* in large trials.

A serious error is to exclude from the statistical analysis any post-randomization, pre-treatment deaths in the active treatment group, while retaining all the untreated controls. The safest general rule is always to leave all randomized patients in (and to randomize later in your next trial!) for in a large trial the overall results will only be materially affected by exclusions if there is a discrepancy between the numbers of exclusions in the two treatment groups which is so marked that it suggests their exclusion was invalid.

### 13D.—Example: retaining deviants.

This has arisen recently in the MRC trial of elective splenectomy in chronic granulocytic leukaemia. Patients with chronic granulocytic leukaemia are randomized to be either splenectomized or not when their disease is in remission. Unfortunately, some of the no-splenectomy group may later develop splenic symptoms which call for splenectomy, and so a few of the patients randomly allocated to no-splenectomy may in fact be splenectomized! Those allocated to the no-splenectomy group who actually seem to need (and therefore get) splenectomy are unlikely to live as long as average, so if we excluded them or if we transferred them from one group to the other group, the 2 groups would no longer be comparable.

The simple comparison of all patients actually splenectomized with all truly non-splenectomized patients is not a valid measure of the value or otherwise of elective splenectomy, as these 2 categories were not separated from each other at random. Neither is a comparison of the group which was randomly allocated for splenectomy with the group which was not splenectomized valid. We must not treat any patient as lost even if that patient has to be splenectomized later on. We must follow all of them to death or to the end of the trial, and then compare the group which was *randomized* to splenectomy with the group which was *randomized* to no-splenectomy, whatever subsequently happened to them. This answers the medical question of primary interest, which is whether a *policy* of splenectomy, if medically possible, is superior to a *policy* of no splenectomy unless specifically indicated.

When departures from protocols are necessary, clinical trials compare a *policy* of one treatment as far as possible with another *policy*, and although it is often of interest to *describe* the results among the protocol adherents, comparisons which omit protocol deviants cannot be tested statistically.

### 14.—*When to analyse and publish your results*

**Early analysis of a trial can be misleading if a temporary difference, which would have been smoothed out by large numbers, causes the trial to be aborted so that large numbers never accumulate.**

Most statistical tests applied to clinical trial data are based on the assumption, usually false, that the decision to stop and publish has been taken completely independently of the current results. This is true of the methods we will describe, and of the methods used in almost all published reports of trials. However, only an investigator with superhuman willpower or completely chaotic records could supervise a clinical trial for months or years without ever looking to see which way the results were drifting, and in practice every now and then at least a cursory impression is usually sought. If no striking difference is apparent, the trial may tick on, but if there is an apparent difference a more formal analysis will be undertaken, and if this is positive, the trial is likely to be stopped and its results published.

Suppose you look every 6 months at a 3-year trial that is comparing treatments which are really equivalent, and ask on each occasion, " Is it significant at the 0·05 level yet? " The chance that it will be on any one particular occasion is 0·05, but unfortunately the chance that it will be on at least one of the 5 occasions is much more than 0·05. If your policy is to look every 6 months and publish if you ever find $P < 0{\cdot}05$, then the chance that you will publish a " significant ($P < 0{\cdot}05$) difference " in a trial comparing 2 treatments which are, in fact, identical is probably more like 15% than the 5% which is claimed, and for this reason, most published $P$-values should be mentally doubled or tripled (McPherson, 1974). Statistical theory can, by standard methods, compute a $P$-value on the assumption that no preliminary examination occurred, and statistical theory can, by " sequential " methods, compute a $P$-value on the assumption that very frequent preliminary examination has occurred, with the intent of stopping the trial when a given difference was reached. (Particular sequential methods for cancer trials are discussed in Statistical Note 4 on p. 611.)

Unfortunately, statistical theory cannot in principle compute a $P$-value from a trial in which occasional preliminary examination occurred: one can only say that the true significance level must be less extreme than the cited $P$-value, but not as much less extreme as it would have been had very frequent preliminary examination occurred.

This present state of affairs is not satisfactory, and no easy universal solution exists. One simple rule, that will often help considerably, is to avoid any analysis (or even brief inspection) of the data until some dozens of deaths have accumulated, for *it is trials first looked at when very small that are most likely to be misleading*. Eventually, the fear that one treatment may be vastly worse will compel inspection, but the longer the delay before first analysis, the smaller the risk of a misleadingly significant difference being discovered when the treatments are really equivalent.

The simplest solution is to continue as at present, where most published $P$-values need to be mentally doubled. The only completely ethical and valid alternative is for every clinical trial to have a professional statistician, using weekly computerized analyses to administer a sequential design. Because the present article is intended to enable clinicians to design and analyse their trial, without getting tied up in statistical knots, we shall ignore the possibility of using sequential methods, (except for those suggested in Statistical Note 4) and shall describe instead the straightforward, non-sequential analysis of ordinary clinical trial data, despite the drawbacks outlined above.

### 15.—*Ethical considerations*

**Individuals must never be denied clearly appropriate treatment, even if trial protocols are thereby disrupted.**

Physicians who are convinced that one treatment is better than another for a particular patient of theirs cannot ethically choose at random which treatment to give: they must do what they think best for the particular patient. For this reason, physicians who feel they already know the answer cannot enter their patients into a trial. If they think, whether for a wise or silly reason, that they know the answer before the trial starts, they should not enter any patients, and if they become convinced that one treatment is better during the course of the trial, they must stop randomizing their patients.

To avoid trials grinding to a halt before any marked degree of statistical significance has been obtained, Chalmers has suggested that it may be necessary to keep the treating physicians ignorant of the current state of the treatment comparison, and only to allow access to the pooled results from all the centres to a small steering committee which decides when the trial shall stop, giving no progress reports whatever on the results until after the trial intake has been halted. (An intermediate alternative, routinely used by Zelen and his colleagues, might sometimes be to give progress reports, with outcome tabulated by treatment, but keeping secret which treatment is which.) The ethical considerations of the supervisory committee can then be guided by slightly wider perspectives than the treating physicians, balancing the damage done by an inconclusive result against the damage done by continuing to allocate some patients to a schedule which appears at the time to be suboptimal. Such a policy has not been adopted by the MRC Leukaemia Steering Committee, partly because so few of our trials have discovered any differences, but we may some day have to do so.

This is not an easy matter: any trial which produces good evidence that one treatment is better than another would have produced almost as good evidence had the last patient given the inferior treatment actually been assigned to the other treatment. If the developing trend is already appreciated by the physician before this last patient is randomized, how can allocation to the inferior treatment be justified? Continuation of this argument suggests that serious consideration of each individual patient's welfare will lead to policies which prevent any clinical trial from producing a clear answer,* which,

* Unless response is so slow that admission of all patients is complete before the results emerge.

paradoxically, will be policies detrimental to the very people they are designed to protect. An ethical imperative exists which is frequently ignored, that we must, if we can, discover how patients can be treated most effectively, and where this requires randomized trials, the apparently irrelevant device of keeping the physicians ignorant of the current results just manages to avert the previous paradox. (Another device which can, in slow diseases such as cancer, help avert it, is to make total entry as rapid as possible, so that entry is finished before any trends can be seen.)

" Blind " trials, where the patient, the doctor, or both, are unaware of which treatment has been given, are frequently easier to organize if placebo treatments are given to the control patients. It is doubtful whether any invasive placebo treatments, involving, for example, dummy injections or infusions, are justifiable, unless a very unusual degree of informed consent has first been given, and the same applies to diagnostic surgery, X-ray investigations, and to bone-marrow (or other) aspirations, if these are of no value to the individual patient.

Finally, of course, it may be ethically necessary not to adhere to a trial schedule which is clearly unsuitable for a particular patient, even though this slightly muddies the scientific comparison of schedules with each other, by diluting one group with people who did not receive the scheduled treatment. In all trials, clinicians must tread a narrow path between unnecessary deviations from protocol and the risks that the protocol may have for the exceptional patient.

M. C. Pike is supported by Contract NO1-CP-53500 and Grant PO ICA 17054-02 from the National Cancer Institute, and N. Mantel by PHS Grant CA 15686.

We are grateful to Gale Mead for typing the annual rewrites which this manuscript has suffered since 1971, and to the many colleagues who used and criticized previous versions.

### REFERENCES

ARMITAGE, P. (1975) *Sequential Medical Trials*. 2nd ed. Oxford: Blackwell Scientific Publications.

BRESLOW, N. E. (1975) Analysis of Survival Data under the Proportional Hazards Model. *Int. statist. Rev.* **43**, 45.

BYAR, D. P., SIMON, R. M., FRIEDEWALD, W. T., SCHLESSELMAN, J. J., DEMETS, D. L., ELLENBERG, J. H., GAIL, M. H. & WARE, J. H. (1976) Randomized Clinical Trials: Perspectives on some New Ideas. *New Engl. J. Med.*, **295**, 74.

CHALMERS, T. C. (1975) Symposium on Diseases of the Liver: Randomization of the First Patient. *Med. Clins N. Am.*, **59**, 1035.

COX, D. R. (1972) Regression Models and Life Tables (with discussion). *J. R. statist. Soc.*, B, **34**, 187.

GEHAN, E. A. (1965) A Generalised Wilcoxon Test for Comparing Arbitrarily Singly-censored Samples. *Biometrika*, **52**, 203.

KAPLAN, E. L. & MEIER, P. (1958) Nonparametric Estimation from Incomplete Observations. *J. Am. statist. Ass.*, **53**, 457.

MANTEL, N. (1966) Evaluation of Survival Data and Two New Rank Order Statistics Arising in its Consideration. *Cancer Chemother. Rep.*, **50**, 163.

MCPHERSON, K. (1974) Statistics: The Problem of Examining Accumulating Data more than Once. *New Engl. J. Med.*, **290**, 501.

PETO, R. & PETO, J. (1972) Asymptotically Efficient Rank Invariant Test Procedures. *Jl R. statist. Soc.*, *A*, **135**, 185.

PETO, R. & PIKE, M. C. (1973) Conservatism of the Approximation $\Sigma(O\text{-}E)^2/E$ in the Logrank Test for Survival Data or Tumor Incidence Data. *Biometrics*, **29**, 579.

PETO, R. (1972) Rank Tests of Maximal Power against Lehmann-type Alternatives. *Biometrika*, **59**, 472.

SMITHSONIAN SCIENCE EXCHANGE CORPORATION, WASHINGTON D.C. (1976) *International Cancer Research Data Bank Program: Compilation of Clinical Protocol Summaries for the National Cancer Institute.*

WILLOUGHBY, M. L. N. (1974) Treatment of Overt Meningeal Leukaemia. *Lancet*. i, 363.

WINDHORST, D. (1976) Principal Investigators and Titles from *Compendium of Tumor Immunotherapy Protocols*. International Registry of Tumor Immunotherapy, National Institutes of Health, Bethesda, Maryland.

### APPENDICES

APPENDIX 1.—*The Relationship Between Numbers and Sensitivity*

It is possible for a clinical trial comparing 2 treatments to produce a " statistically significant " ($P < 0{\cdot}05$) difference even if the treatments are, in fact, equivalent in their effects. A far more common mishap is for a comparison of 2 treatments which really are different to fail to reach statistical significance.

However, if there really is a difference between the chances of survival under the 2 treatments, the more patients we put into the trial the more likely we are to get a statistically significant difference, and this Appendix indicates the approximate numbers of patients that must be randomized to have an even chance of a statistically significant outcome when the trial is finally analysed. Of course, the numbers required depend on how big the true difference is, and if you knew *that* at the design stage you would not be doing the trial anyway! However, in practice, physicians usually have an idea of what differences might well exist between 2 treatments, and this Appendix is intended to be used to ensure that ridiculously small trials are not started (see also main text). Unless you can say " such-and-such a difference *might well* exist, and if it does we have a better than even chance of detecting it ", you should not start a clinical trial.

Approximately equivalent numbers are usually needed for a null trial result to be useful. Suppose that, in a trial sufficiently large for a *given difference* to have an even chance of being detected, the 2 groups eventually fare about the same as each other. You will be able to conclude (with " 95% confidence ") that the real difference could be zero and could be anything up to that *given difference* in either direction, but that the real difference could hardly be any more extreme than this. If, therefore, that *given difference* was one which you had previously thought *might well* exist, your null trial has told you something new, but otherwise it has not.

To use this Appendix, first estimate roughly how many patients you will be able to randomize, and roughly how many of these will die before the trial is analysed. This is because the sensitivity of a clinical trial depends not so much on the total number of patients randomized into the trial, but rather on the number of patients who die before the statistical analysis takes place. Next, refer this projected number of deaths to Table II to see what magnitude of treatment differences you are competent to characterize. The calculations leading to this table need not be understood, but the conclusions it expresses must be.

For a given degree of superiority of one treatment over the other, Table II indicates the number of patients that must die in a clinical trial for there to be an even chance of a statistically significant difference being observed. Even if the observed difference is not statistically significant, however, it is still likely to be in the right direction (and likely to be nearer being significant than to being zero) and thus, it will probably *suggest* the correct answer. If the trial size is doubled, the chance of statistical significance rises from 50% to 80%, and even if statistical significance is, unfortunately, missed, there is almost bound to be a substantial difference in the right direction.

*Example*—If we expect an average intake of 2 acute myeloid leukaemia patients a week, and we expect about half the patients to die within 6 months of entry, half the survivors to die within the next 6 months, etc., then what sensitivity could we expect from a clinical trial, the intake to which lasted one year?

*Answer*—By the time entry is complete, one year after entry started, about 100 patients will have been randomized, and we might find that about 40 of these patients will already be dead. If we analyse immediately, column (b) of Table II tells us that we have an even chance of detecting an improvement from 50% dead within 6 months of randomization to 30% dead within 6 months, but that we are unlikely to detect any less striking differences. If, however, we delay our analysis for a year or two, until most of our patients have died, we will have observed about 100 deaths and would have an even chance of detecting an improvement from 50% dead within 6 months of randomization to 37% dead. Again, however, any less extreme improvements are likely to elude us.

### APPENDIX 2.—*Detailed Instructions for Preparing " Balanced " Randomization Lists*

Balancing the allocation numbers at intervals ensures that no time trend in the allocation ratio will exist, and so no bias can arise, even if a time trend in the prognoses of the patients exists for any reason.

One way of pseudo-randomizing in the ratio 1:1 is to make sure that the randomization balances at every 6th patient. This can be done by numbering the 20 possible different sequences of 3 A's and 3 B's as follows:

| Number | Sequence | Number | Sequence | Number | Sequence | Number | Sequence |
|---|---|---|---|---|---|---|---|
| 00–04 | AAABBB | 25–29 | ABABAB | 50–54 | BAAABB | 75–79 | BABBAA |
| 05–09 | AABABB | 30–34 | ABABBA | 55–59 | BAABAB | 80–84 | BBAAAB |
| 10–14 | AABBAB | 35–39 | ABBAAB | 60–64 | BAABBA | 85–89 | BBAABA |
| 15–19 | AABBBA | 40–44 | ABBABA | 65–69 | BABAAB | 90–94 | BBABAA |
| 20–24 | ABAABB | 45–49 | ABBBAA | 70–74 | BABABA | 95–99 | BBBAAA |

You then select one 2-digit number at random (*e.g.* by shutting your eyes, marking the Random Number Table (Table III) haphazardly with a pencil point, and then starting with the number nearest to the mark thus made), and read off the series of 2-digit numbers from there on. Replace each such 2-digit number by the corresponding letter sequence and use the overall sequence of A's and B's thus generated to specify the order of the contents of your randomization envelopes.

A similar approach to 3-group trials is possible; in order to get a random ordering of 2 A's, 2 B's and 2 C's, proceed as above but, having selected one of the above sequences, use the *next* 2-digit random number to change one of the A's and one of the B's into C's, as follows:

| Next no.* | Change into C | Next no. | Change into C | Next no. | Change into C |
|---|---|---|---|---|---|
| 01–11 | First A, First B | 34–44 | Second A, First B | 67–77 | Third A, First B |
| 12–22 | First A, Second B | 45–55 | Second A, Second B | 78–88 | Third A, Second B |
| 23–33 | First A, Third B | 56–66 | Second A, Third B | 89–99 | Third A, Third B |

* If 00 arises, ignore it and proceed to the next 2-digit random number.

Finally, a similar approach to randomization in the ratio 2:1 or 1:2 is possible: simply produce a balanced sequence of A's, B's and C's as above, and then either change all the C's to A's (for a 2:1 sequence) or change all the C's into B's (for a 1:2 sequence).

*Example*—If the mark made by your pencil is nearest to the number 33 in the 3rd row of Table III, the numbers obtained will be 33, 16, 26, 91, 57, 58, etc., giving:

| Random Number | 33 | 16 | 26 | 91 | 57 | 58 | ... |
|---|---|---|---|---|---|---|---|
| 1:1 Sequence | ABABBA<br>~~A~~BA~~B~~BA<br>C C | AABBBA | ABABAB<br>ABAB~~A~~~~B~~<br>CC | BBABAA | BAABAB<br>BA~~A~~BA~~B~~<br>C C | BAABAB | ... |
| 1:1:1 Sequence | | CBACBA | | ABABCC | | BACBAC | ... |
| 2:1 Sequence | | ABAABA | | ABABAA | | BAABAA | ... |
| 1:2 Sequence | | BBABBA | | ABABBB | | BABBAB | ... |

If you have to prepare one randomization list for each of several centres, the easiest way is to produce a single list of the appropriate length for one centre, and then to start at a different place on it for each different centre, returning to the top of the list again whenever you reach the bottom of it.

*Multiple randomization* (*answering* 2 *or more questions with one clinical trial*). Suppose you wish to answer 2 questions, one of which is answered by comparing treatment A with treatment B, the other question being answered by comparing treatment $\alpha$ with treatment $\beta$. Prepare 3 independent, separate lists: one for randomizing A versus B, the second for randomizing the A's between $\alpha$ and $\beta$, and the third for randomizing the B's between $\alpha$ and $\beta$. If the decision between $\alpha$ and $\beta$ does not need to be taken until some time after the decision between A and B has been taken (*e.g.* if A and B relate to induction, while $\alpha$ and $\beta$ relate to maintenance) keep the lists separate, and do not randomize between $\alpha$ and $\beta$ until you have to. If the decisions all need to be specified at the same time, the lists can all be combined (by running down the A's on the first list and writing $\alpha$ or $\beta$ beside each according to the second list, and

TABLE II.—*Approximate Sensitivity of Clinical Trials with a Given Total Number of Deaths at the Time of Analysis of the Results, among Patients Randomized Equally between 2 Treatments*

| Total no. of patients already dead at the time we analysed the trial. (Far more patients must be randomized to observe these numbers) | Approximate Conditions for an Even Chance of Obtaining a Statistically Significant Result: (a) If less than half the patients will die and the ratio of the death rate on the better treatment to that on the worse is as stated below:* Better:Worse | (b) If half or more of the patients will die, and the chance of death on the better treatment by the time when half the patients on the worse treatment are dead is as stated below:* |
|---|---|---|
| 1200 | 8:9 | 0·46 |
| 500 | 5:6 | 0·44 |
| 200 | 3:4 | 0·40 |
| 100 | 2:3 | 0·37 |
| 40 | 1:2 | 0·3 |
| 20 | 1:3 | 0·2 |
| 10 | 1:6 | 0·1 |

* For a mathematical definition of columns (a) and (b), see Statistical Note 5 on p. 612.

then running down the B's according to the third list). If 3 questions are being answered a further 4 extra lists must be prepared, randomizing between the third pair of treatments for each of A$\alpha$, A$\beta$, B$\alpha$, and B$\beta$. If treatment A really is better than treatment B, its relative merits will by chance show up more clearly either among the $\alpha$-treated patients or among the $\beta$-treated patients, and balancing the proportion of $\alpha$'s and $\beta$'s in groups A and B helps ensure that a simple benefit of A is not confused with a benefit of A only if $\alpha$ is also given.

### STATISTICAL NOTES

These are collected together so that they can be completely ignored by the non-statistical reader. The statistical methods recommended in this paper are developed in Kaplan and Meier (1958), Mantel (1966), Peto (1972), Cox (1972), and Peto and Pike (1973), and are reviewed by Breslow (1975).

### STATISTICAL NOTE 1.

(From Table I.) Most investigators have a few cursory glances (or even full analyses) comparing their 2 treatment groups while the trial progresses, and if a significant difference is noticed the trial is likely to be stopped and published. This leads to some trials, which would have been non-significant if interim inspection had been avoided, actually being published as smaller "significant" results. Such practice increases the number of *misleading* claims of significance by a greater

TABLE III.—*Random Number Table*

| | | | | | | | | | | | | | | | | | | | |
|---|---|---|---|---|---|---|---|---|---|---|---|---|---|---|---|---|---|---|---|
| 71 | 11 | 41 | 82 | 79 | 37 | 00 | 45 | 98 | 54 | 52 | 89 | 26 | 34 | 40 | 13 | 60 | 38 | 08 | 86 |
| 61 | 05 | 66 | 18 | 76 | 82 | 11 | 18 | 61 | 90 | 90 | 63 | 78 | 57 | 32 | 06 | 39 | 95 | 75 | 94 |
| 81 | 89 | 42 | 34 | 00 | 49 | 97 | 53 | 33 | 16 | 26 | 91 | 57 | 58 | 42 | 48 | 51 | 05 | 48 | 27 |
| 10 | 24 | 90 | 84 | 22 | 16 | 26 | 96 | 54 | 11 | 01 | 96 | 58 | 81 | 37 | 97 | 80 | 98 | 72 | 81 |
| 14 | 28 | 33 | 43 | 01 | 32 | 58 | 39 | 19 | 54 | 56 | 57 | 23 | 58 | 24 | 87 | 77 | 36 | 20 | 97 |
| 35 | 41 | 17 | 89 | 87 | 04 | 28 | 32 | 13 | 45 | 59 | 03 | 91 | 08 | 69 | 24 | 84 | 44 | 42 | 83 |
| 07 | 89 | 36 | 87 | 98 | 73 | 77 | 64 | 75 | 19 | 05 | 61 | 11 | 64 | 31 | 75 | 49 | 38 | 96 | 60 |
| 27 | 59 | 15 | 58 | 19 | 68 | 95 | 47 | 25 | 69 | 11 | 90 | 26 | 19 | 07 | 48 | 83 | 59 | 90 | 95 |
| 95 | 98 | 45 | 52 | 27 | 35 | 86 | 81 | 16 | 29 | 37 | 60 | 39 | 35 | 05 | 24 | 49 | 00 | 29 | 07 |
| 12 | 95 | 72 | 72 | 81 | 84 | 36 | 58 | 05 | 10 | 70 | 50 | 31 | 04 | 12 | 67 | 74 | 01 | 72 | 90 |
| 35 | 23 | 06 | 68 | 52 | 50 | 39 | 55 | 92 | 28 | 28 | 89 | 64 | 87 | 80 | 00 | 84 | 53 | 97 | 97 |
| 86 | 33 | 95 | 73 | 80 | 92 | 26 | 49 | 54 | 50 | 41 | 21 | 06 | 62 | 73 | 91 | 35 | 05 | 21 | 37 |
| 02 | 82 | 96 | 23 | 16 | 46 | 15 | 51 | 60 | 31 | 55 | 27 | 84 | 14 | 71 | 58 | 94 | 71 | 48 | 35 |
| 44 | 46 | 34 | 96 | 32 | 68 | 48 | 22 | 40 | 17 | 43 | 25 | 33 | 31 | 26 | 26 | 59 | 34 | 99 | 00 |
| 08 | 77 | 07 | 19 | 94 | 46 | 17 | 51 | 03 | 73 | 99 | 89 | 28 | 44 | 16 | 87 | 56 | 16 | 56 | 09 |
| 61 | 59 | 37 | 08 | 08 | 46 | 56 | 76 | 29 | 48 | 33 | 87 | 70 | 79 | 03 | 80 | 96 | 81 | 79 | 68 |
| 67 | 70 | 18 | 01 | 67 | 19 | 29 | 49 | 58 | 67 | 08 | 56 | 27 | 24 | 20 | 70 | 46 | 31 | 04 | 32 |
| 23 | 09 | 08 | 79 | 18 | 78 | 00 | 32 | 86 | 74 | 78 | 55 | 55 | 72 | 58 | 54 | 76 | 07 | 53 | 73 |
| 89 | 40 | 26 | 39 | 74 | 58 | 59 | 55 | 87 | 11 | 74 | 06 | 49 | 46 | 31 | 94 | 86 | 66 | 66 | 97 |
| 84 | 95 | 66 | 42 | 90 | 74 | 13 | 71 | 00 | 71 | 24 | 41 | 67 | 62 | 38 | 92 | 39 | 26 | 30 | 29 |

proportion than the number of *right* claims is increased, and it affects the reliability of claims of statistical significance in reports of *small* (*i.e.* curtailed) trials much more than in reports of *large* trials (*i.e.* nearing completion). This accentuates the conclusion, already suggested by the last column in Table I, that significant results in small trials are often wrong.

The above considerations apply to that large majority of studies in which there is no real difference or only a moderate real difference, so small that it is likely to be missed by a small trial. However, in those few studies which compare treatments so enormously different that a small trial is likely to detect this, opposite conclusions apply: such trials will necessarily stop when still small, and such differences will therefore only be found among significant small trials. Proper treatment of this whole question requires that a distribution of real differences be postulated, and its consequences considered; as long as this postulated distribution has a large majority of real differences so small that they are unlikely to be detected by small trials, the conclusions stated in the main text remain valid.

STATISTICAL NOTE 2.

(From p. 600.) An alternative sometimes suggested is to vary the probability of allocation of the next patient to each treatment, in the light of the data so far, to make allocation to the apparently better treatment progressively more probable. If, however, there is a tendency for later patients to fare better irrespective of treatment, then data-dependent allocation will produce too many extreme $P$-values comparing treatments which do not really differ, while if the opposite time trend exists, a real superiority of one treatment may be masked. The much larger disadvantage is the possibility of conscious or subconscious cheating in the selection of patients for the trial. At the end of such a trial, the physicians will know that their favourite treatment is doing well, that most new trial patients will now receive it, and that if just a few more patients put on it now do well, the trial will appear conclusive. Severe bias is an obvious possibility, either for the treatment or, leaning over backwards to be fair, against it.

Another disadvantage is that such designs may not be as acceptable to physicians as simple randomization is, partly because the underlying ideas will not be clearly understood and partly because it may prove difficult to convince some physicians, worried about mistreatment of their patients, that the current results justify a substantial decrease in the proportion allocated to the seemingly inferior treatment but do not justify its abandonment.

STATISTICAL NOTE 3.

(From p. 601.) Statistical power depends on the triple product of the size of a stratum and the proportions on the 2 treatments. These proportions may either be fixed or binomial; the product, if they are fixed, equals the expected product, if they were binomial in a stratum containing one extra patient.

STATISTICAL NOTE 4.

(From p. 605.) Sequential use of the logrank test is described in Chapter 7 of the 2nd edition (1975) of *Sequential Medical Trials* by P. Armitage. (The 1st edition does not contain such methods.) The advantage of sequential analysis is the guarantee that in the rare cases when one treatment is *much* worse, the trial will automatically be aborted. However, the advantage of not looking at the results until the last minute is that if, as is more usual, one treatment is only *moderately* worse (*e.g.* if the better treatment only reduces the death rate by 50% or less), a statistically significant difference is more likely to emerge than if sequential methods had been used.

These advantages might be combined by a policy of not stopping prematurely, unless the chi-square comparing treatments reaches some rather extreme value such as 9 (referring to Armitage's " Repeated Significance Test " designs for the $P$-value if it does). Otherwise, let the trial run on to a previously agreed final size or date, when ordinary statistical analysis is undertaken. (Correction of the final $P$-value for the stopping policy is possible, but would usually be unnecessary.) This policy would be peculiarly appropriate for cancer trials, where prolonged follow-up is usual: here, the monitoring would usually be needed only while new patients were still being randomized, since, once this phase is complete, the ethical difficulties of continuing to accumulate follow-up information may be less acute (unless patients could still, with advantage, be switched to the superior treatment late in the course of the disease).

STATISTICAL NOTE 5.

(From Table II.) The death rate among the survivors of a group will in general vary according to how long ago randomization occurred (since an exactly exponential distribution of survivorship is unusual). If, in 2 groups, the death rates vary in parallel, that in one group among the survivors at a given time after randomization being a constant multiple $\lambda$ of the corresponding rate in the other group, then we have a " Proportional Hazard " situation (Breslow, 1975). If a 2-tailed 95% significance test of the null hypothesis is performed between 2 such groups, using a logrank test, it is more likely to be significant if $\lambda$ is markedly different from unity or if the numbers are large. Table II quantifies this: if, in column (a), the ratio of Better/Worse is $\lambda$, column (b) gives $1-(0 \cdot 5)^{\lambda}$.

If $\lambda$ is constant and is approximately, but not exactly, unity, then the logrank test is more likely to detect the difference between the 2 groups than any other unbiased rank-invariant test procedure is, and is asymptotically fully efficient. (By contrast, Peto and Peto (1972) noted that no class of differences exists against which Gehan's (1965) generalization of the Wilcoxon test is asymptotically efficient.) Of course, even if the Proportional Hazard is not appropriate, the logrank test is still a valid test of the null hypothesis.